# Elucidating the Reactivity of Oxygenates on Single-Atom Alloy Catalysts

**DOI:** 10.1021/acscatal.3c03954

**Published:** 2023-11-27

**Authors:** Weitian Li, Simran Effricia Madan, Romain Réocreux, Michail Stamatakis

**Affiliations:** † Thomas Young Centre and Department of Chemical Engineering, University College London, Roberts Building, Torrington Place, London WC1E 7JE, U.K.; ‡ Yusuf Hamied Department of Chemistry, University of Cambridge, Lensfield Road, Cambridge CB2 1EW, U.K.

**Keywords:** single-atom alloy, catalyst screening, linear scaling, Bro̷nsted−Evans−Polanyi relation, methanol dissociation, DME synthesis

## Abstract

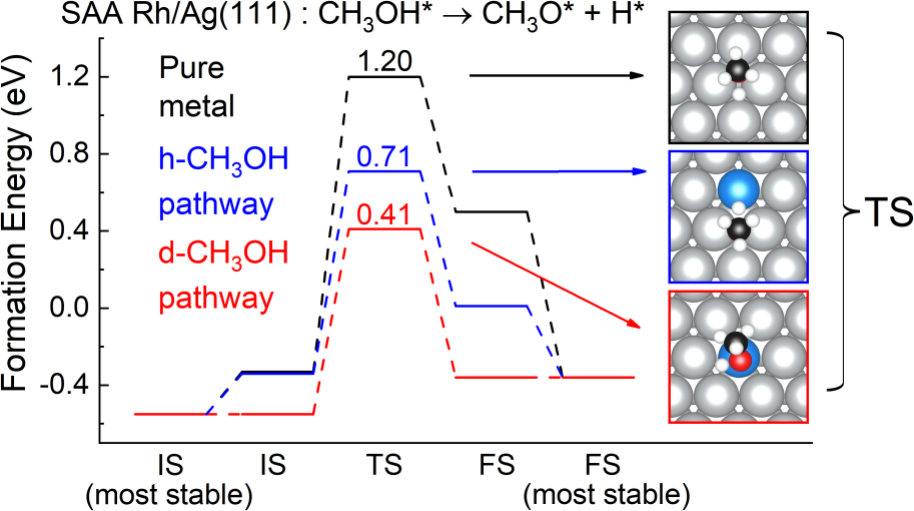

Doping isolated transition metal atoms into the surface of coinage-metal hosts to form single-atom alloys (SAAs) can significantly improve the catalytic activity and selectivity of their monometallic counterparts. These atomically dispersed dopant metals on the SAA surface act as highly active sites for various bond coupling and activation reactions. In this study, we investigate the catalytic properties of SAAs with different bimetallic combinations [Ni-, Pd-, Pt-, and Rh-doped Cu(111), Ag(111), and Au(111)] for chemistries involving oxygenates relevant to biomass reforming. Density functional theory is employed to calculate and compare the formation energies of species such as methoxy (CH_3_O), methanol (CH_3_OH), and hydroxymethyl (CH_2_OH), thereby understanding the stability of these adsorbates on SAAs. Activation energies and reaction energies of C–O coupling, C–H activation, and O–H activation on these oxygenates are then computed. Analysis of the data in terms of thermochemical linear scaling and Bro̷nsted–Evans–Polanyi relationship shows that some SAAs have the potential to combine weak binding with low activation energies, thereby exhibiting enhanced catalytic behavior over their monometallic counterparts for key elementary steps of oxygenate conversion. This work contributes to the discovery and development of SAA catalysts toward greener technologies, having potential applications in the transition from fossil to renewable fuels and chemicals.

## 1 Introduction

Searching for efficient and sustainable energy sources and carriers as well as “green” chemicals manufacturing processes are critical challenges that contemporary societies are facing. Converting CO_2_ to renewable energy sources is a promising way to lower the carbon footprint and mitigate the impacts of global warming. Biomass reforming is also an important process that converts biomass into useful fuels and chemicals and has the potential to provide a sustainable alternative to traditional fossil fuel processing. Heterogeneous catalysis is envisioned to play a crucial role in facilitating the reactions necessary for biomass conversion, and the development of new catalysts is key to achieving these goals.^1,2^

Single-atom catalysts have become the focal point in heterogeneous catalysis after being proposed in 2011.^3^ Since then, both theoretical analyses and experimental results have demonstrated their potential for high stability, selectivity, activity, and cost-effectiveness.^4−6^ Single-atom alloys (SAAs), as a type of single-atom catalysts, formed by doping isolated transition metal atoms into the surface of coinage metal hosts, have emerged as promising catalysts that can combine the activity and selectivity of transition and coinage metals in a balanced way.^7−9^ Recent studies have demonstrated the unique electronic structure of SAAs, with a very sharp d-band feature close to the Fermi level, which can be used to tune their adsorption properties.^10^ Moreover, by appropriately choosing bimetallic combinations, the electronic structure of SAAs can be tailored for enhanced selectivities and activities for given reactions.^10,11^

Specifically for reactions involving oxygenates, the outstanding performance of SAAs has been widely confirmed by both theoretical and experimental research. For instance, regarding methanol (CH_3_OH) synthesis, Pd, Rh, Pt, and Ni are able to promote the production of CH_3_OH from CO_2_ on Cu(111), where the conversion via the reverse water gas shift reaction is followed by a CO hydrogenation pathway.^12^ Ru/Co and Ru/Pt surfaces also exhibit enhanced catalytic activities compared to pure Ru due to the synergistic effects of weak binding and improved charge redistribution.^13^ In addition, Rh/Cu SAAs can facilitate the production of CH_3_OH from syngas (CO + H_2_).^14^ Additionally, the Pt/Cu and Pd/Cu SAAs exhibit outstanding selectivity and activity when used to dehydrogenate CH_3_OH to formaldehyde (CH_2_O).^15,16^ For ethanol dehydrogenation, Ni/Cu, Pt/Cu, Pd/Au, Rh/Au, and Ni/Au SAAs were demonstrated to have better selectivity and/or higher activity compared to their pure host metals.^17−25^

These results indicate that SAAs have the potential to be superior catalysts in comparison to pure metals for biomass reforming. However, discovering metal combinations that would act as high-performance catalysts for given systems is not an easy task. In most cases, catalytic materials are discovered through expensive and time-consuming trial and error methods.

To solve this problem, theory and simulation can facilitate catalyst discovery studies. Computational investigations of the electronic structure of large molecules, crystals, and other complex structures currently adopt density functional theory (DFT) as a practical quantum mechanical modeling technique.^26−29^ Due to its high computational cost for large systems, however, the effectiveness of this method in catalyst discovery is limited, and efforts have been concentrated on developing descriptor-based models from a subset of DFT data to reduce this computational burden.^27−29^

In catalyst design, thermochemical scaling (TCS) and Brønsted–Evans–Polonyi (BEP) relationship are two prevalent descriptor-based models. TCS^28−30^ describes the linear relationship between the adsorption energies of similar adsorbates, which enables adsorption energy estimations on a large range of adsorbates from a small set of species. The BEP^30−34^ relationship suggests that the reaction energy is linearly related to the activation energy in reactions of the same family. By combining TCS and BEP relationship, within the context of the Sabatier principle, the performance of a set of catalysts for a reaction can be predicted, making descriptor-based models important in catalyst design. In an effort to test these principles on SAAs, Darby et al.^35,36^ performed DFT calculations for H–H, C–H, N–H, O–H, and C–O activations on SAAs and compared the performance of these materials with those of pure metals. It was thus revealed that SAA behavior deviates from linearity, thereby “breaking” the BEP relation and resulting in these materials exhibiting an enhanced catalytic performance. However, these studies were limited to small molecules and can be extended to larger molecules of interest for biomass conversion.

Thus, the present study investigates the catalytic properties of 12 SAAs [including Ni-, Pd-, Pt-, and Rh-doped Cu(111), Ag(111), and Au(111)] for chemistries involving oxygenates that are relevant to biomass reforming. The dehydrogenation of CH_3_OH toward CH_2_O is chosen to investigate C–H and O–H activation, while dimethyl ether (CH_3_OCH_3_) formation from methyl (CH_3_) and methoxy (CH_3_O) is chosen to investigate C–O coupling. Formation energies are first calculated from DFT to explore the validity of TCS relations on these surfaces. To understand the reactivity of these SAAs, we employ DFT to compute the activation and reaction energies of key elementary steps in oxygenate conversion, including C–O coupling, C–H activation, and O–H activation. After that, BEP relationships for each of the aforementioned elementary steps are studied, thereby providing valuable insights into the relationship between the reaction energetics and the catalytic properties of the SAAs.

## 2 Computational Details

### 2.1 Methodology

In this project, DFT calculations are performed using VASP 5.4.1.^37−39^ The van der Waals exchange–correlation functional optB86b-vdW is chosen to calculate heterogeneous systems accurately.^40−43^ Projected augmented wave potentials are used to alleviate computational efficiency challenges caused by core electrons.^44,45^ For the valence electron expansion, the cutoff energy is set to 400 eV. When surface configurations are optimized, the convergence criterion of the ionic step is set to 10^–7^ eV, and the Hellmann–Feynmann forces on all free atoms should be lower than 0.01 eV/Å.

**Figure 1:**
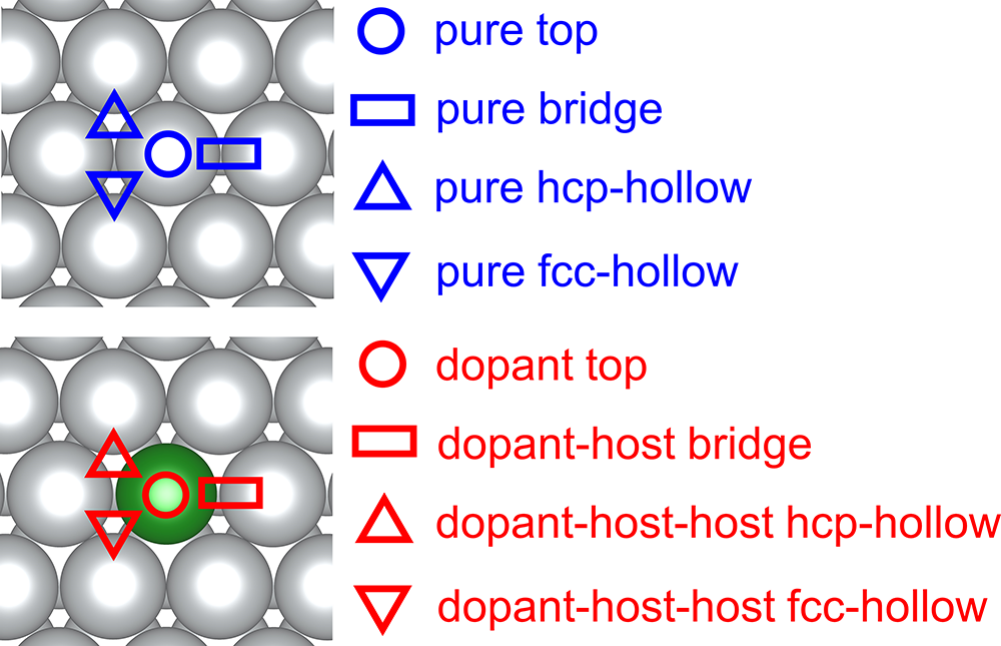
Typical pure metal fcc(111) surface (top, blue symbols and font) and SAA fcc(111) surface (bottom, red symbols and font).

A 5-layer *p*(4 × 4) supercell with the top three layers relaxed and bottom two layers fixed was used to model fcc(111) surfaces for Au and Au-based SAAs, Ag and Ag-based SAAs, Cu and Cu-based SAAs and Ni, Rh, Pt, and Pd catalysts (Figure 1). Lattice constants for the bulk pure fcc metals are the same as in the previous paper (Table S1 in the Supporting Information).^35^ Every supercell has a ∼14 Å vacuum layer to avoid the interaction between periodic images along the direction vertical to the surface.^46^ To achieve a high computational accuracy, the Brillouin zone is sampled with a 9 × 9 × 1 Monkhorst–Pack *k*-point grid.^47^ Stable structures are identified by the conjugate gradient method. Transition states are searched by the climbing image nudged elastic band method or the dimer method and further refined with the quasi-Newton method.^48−50^ Vibrational calculations are then performed to validate these states by ensuring that each transition state has only one imaginary frequency. To verify the initial and final states of each elementary step, the normalized vector of the unstable mode is multiplied by 0.2 and added to (or subtracted from) the validated transition state to obtain two perturbed structures. These perturbed structures are then optimized by the conjugate gradient method to check whether the resulting initial and final states are reasonable.

### 2.2 Energy Definitions

In our screening study, we first focused on the computation of the adsorption geometries and energies of various catalytically relevant adsorbates on SAA fcc(111) surfaces. To investigate the preferable sites of adsorbates on the various SAAs of interest in this work, the energies of the clean surface and the adsorbates were first computed using VASP, followed by calculating formation energies by the following equation:
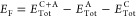
1where *E*_F_ is the formation energy, *E*_Tot_^C+A^ is the total energy of the catalyst and the adsorbate, *E*_Tot_^C^ is the total energy of the catalyst, and *E*_Tot_^A^ is the energy calculated as a linear combination of gas-phase DFT total energies for reference species whose atoms constitute A. In our work, CH_4_, H_2_, and CO_2_ are taken as gas-phase reference species. For example, when A = CH_3_, the formation energy *E*_Tot_^A^ is calculated by
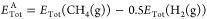
2

After that, the energy barriers and reaction energies for the subsequent steps are calculated. The process of CH_3_OH dehydrogenation to CH_2_O involves several reaction intermediates, including CH_3_OH, hydroxymethyl (CH_2_OH), CH_3_O, and CH_2_O. This study further investigates the reactivity of CH_3_O with CH_3_ as this is always seen as one of the key elementary steps for the formation of CH_3_OCH_3_ (Table 1).

**Table 1:** Reaction Steps of CH_3_OH Dehydrogenation to CH_2_O and Formation of CH_3_OCH_3_

elementary steps	
CH_3_OH* → CH_2_OH* + H*	(A)
CH_2_OH* → CH_2_O* + H*	(B)
CH_3_OH* → CH_3_O* + H*	(C)
CH_3_O* → CH_2_O* + H*	(D)
CH_3_O* + CH_3_* → CH_3_OCH_3_*	(E)
CH_3_O* + CH_3_* → CH_3_OCH_2_* + H*	(F)

Activation and reaction energies for elementary steps were calculated by the following equations:
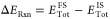
3
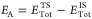
4where Δ*E*_Rxn_ is the reaction energy, *E*_A_ is the activation energy, *E*_Tot_^FS^ is the energy of the final state, *E*_Tot_^IS^ is the energy of the initial state, and *E*_Tot_^TS^ is the energy of the transition state. In contrast to what happens on pure metals, bond scission and coupling on SAAs can occur on dopant and host atoms, leading to different reaction pathways and various energy barriers. Note that at least two reaction pathways for each kind of elementary step, which start from different initial states, are investigated in this work. In this case, the apparent activation energy defined by subtracting the energy of the transition state from that of the most stable initial state can be used to compare different pathways. Lower activation energies are usually associated with high catalytic activities.

## 3 Results and Discussion

### 3.1 Adsorption Configurations and Energies

We first investigate the adsorption geometries and energies of simple organic oxygenates, including CH_3_OH, CH_3_O, CH_3_OCH_3_, CH_3_OCH_2_, CH_2_OH, and CH_2_O, as well as inorganic fragments H_2_O and OH, on the surface of pure metals and SAAs (Table S2). Among these fragments, surface configurations of H_2_O, OH, and CH_3_OH on SAAs were acquired from our previous studies^35,36^ and reoptimized with the computational setup of this work toward a consistent data set. The formation energies were then used to compare the stability of the adsorbates on various SAAs and pure metals.

#### 3.1.1 Preferable Surface Configurations of Each Fragment

On pure transition metals, the most stable sites for the adsorption of H_2_O, CH_3_OH, CH_2_OH, CH_3_OCH_2_, and CH_3_OCH_3_ are the top sites, while CH_3_O and OH prefer to adsorb to the host fcc-hollow sites on these surfaces (Figure S1 in the Supporting Information). Additionally, the adsorption geometries of CH_2_O on pure metal surfaces are more complex. CH_2_O lies far from the surface with its molecular plane perpendicular to the surface when adsorbing on Au(111) and Ag(111).^51,52^ On the other hand, CH_2_O binds to the pure-top site, where C forms bonds to two H, one O, and one surface atom, while the O atom bonds to the neighbor bridge site on the surfaces of other pure metals, in line with previous studies (Figure S2).^53−57^

**Figure 2:**
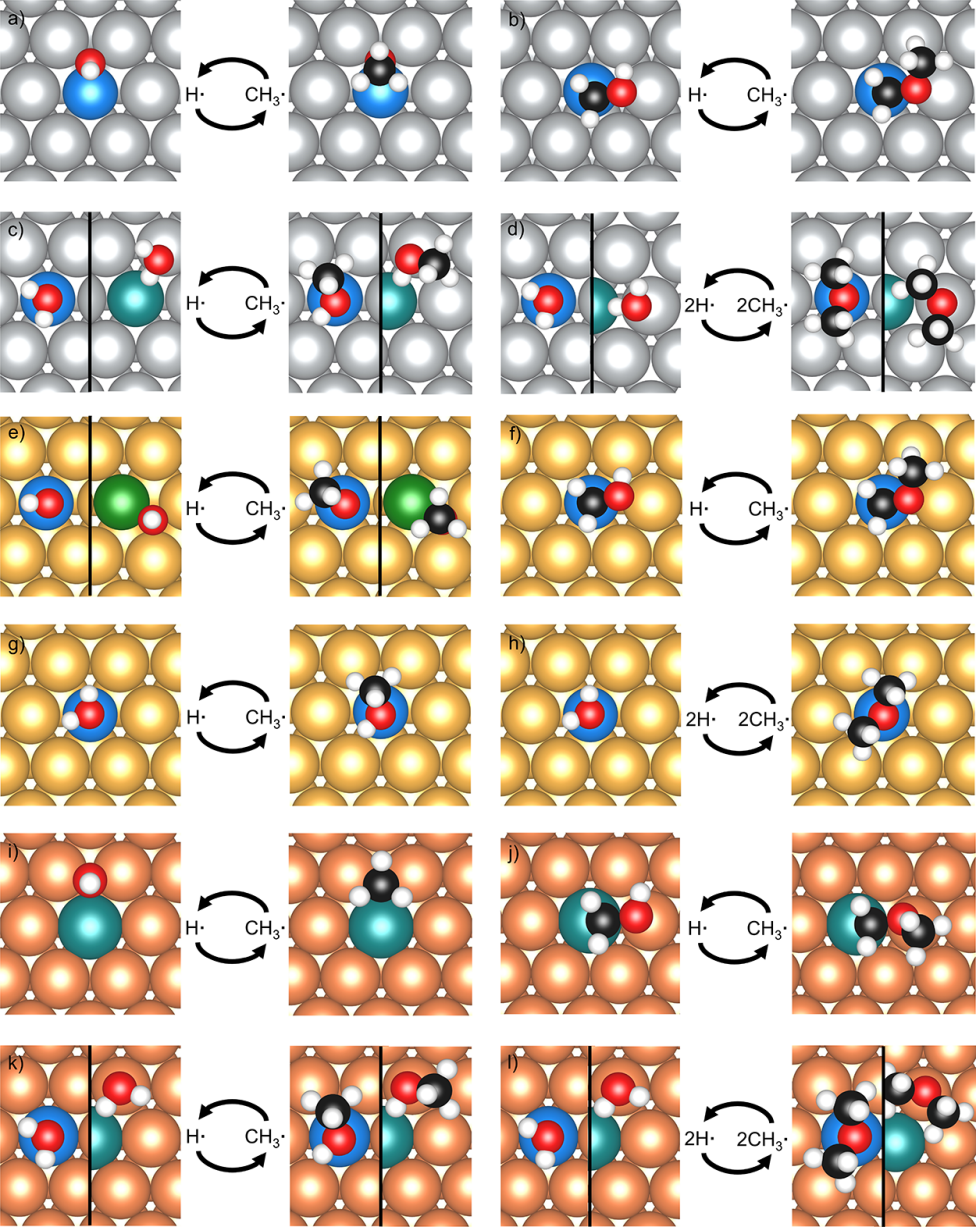
DFT-optimized structures for (a) OH and CH_3_O intermediates and (b) CH_2_OH and CH_3_OCH_2_ intermediates on Rh/Ag(111); (c) H_2_O and CH_3_OH and (d) H_2_O and CH_3_OCH_3_ intermediates on Rh/Ag(111) and Pt/Ag(111); (e) OH and CH_3_O intermediates on Rh/Au(111) and Pd/Au(111); (f) CH_2_OH and CH_3_OCH_2_ intermediates, (g) H_2_O and CH_3_OH intermediates, and (h) H_2_O and CH_3_OCH_3_ intermediates on Rh/Ag(111); (i) OH and CH_3_O intermediates and (j) CH_2_OH and CH_3_OCH_2_ intermediates on Pt/Cu(111); (k) H_2_O and CH_3_OH and (l) H_2_O and CH_3_OCH_3_ intermediates on Rh/Cu(111) and Pt/Cu(111).

On SAAs, CH_3_O prefers to adsorb on host–dopant–dopant fcc-hollow sites via the O atom except for Pt/Au(111) and Rh/Au(111), where the top sites are the most suitable ones exhibiting the lowest formation energies (Figure 2a,e,i). Interestingly, OH, another group that shares an adsorption pattern similar to that of CH_3_O, also exhibits the same exceptions on Pt/Au(111) and Rh/Au(111) (Figure 2e). CH_3_OCH_2_ and CH_2_OH adsorptions are preferable on dopant top sites of every SAA since the C atoms undergo sp^3^ hybridization and bind to the dopant atoms of the SAAs (Figure 2b,f,j). H_2_O, CH_3_OH (Figure 2c,g,k), and CH_3_OCH_3_ (Figure 2d,h,i) bind on the dopant top sites via their O atoms on most SAA surfaces. One exception is that H_2_O and CH_3_OH species are more prone to adsorbing on the top site of the nearest host atoms on Pt/Ag(111), Pt/Cu(111), and Pd/Cu(111) (Figure 2c,k). Similarly, CH_3_OCH_3_ fragments prefer binding on the nearest host atoms on Pt/Ag(111) and Pt/Cu(111) (Figure 2d,i). A possible explanation is that the dopant atoms of Pt/Ag(111), Pt/Cu(111), and Pd/Cu(111) exhibit large negative charges (−0.61, −0.45, and −0.30 *e*, respectively) compared to other SAAs, and thus, these adsorbates prefer staying away from the negatively charged O atoms of H_2_O, CH_3_OH, and CH_3_OCH_3_ (Figure 2c,d,k,i).^58^ CH_2_O binds to the dopant top sites on Pd/Au(111), Rh/Au(111), Ni/Au(111), Ni/Ag(111), and Rh/Ag(111) via the O and C atoms, while on other SAAs, C and O bind to the dopant site and neighbor pure bridge sites, respectively. The adsorption behavior of species investigated in this study follow simple valency rules; thus, closed-shell Lewis bases and species with a single dangling bond prefer to adsorb on the dopant atom top site, while fragments with two or three dangling bonds are most stable on shared dopant–host bridge and dopant–host–host hollow sites, respectively, as previously proposed by Michaelides and Hu.^59^ In the following section, the formation energies for various adsorbates on their most stable sites are compared.

#### 3.1.2 Formation Energy Comparisons and TCS Relations

**Figure 3:**
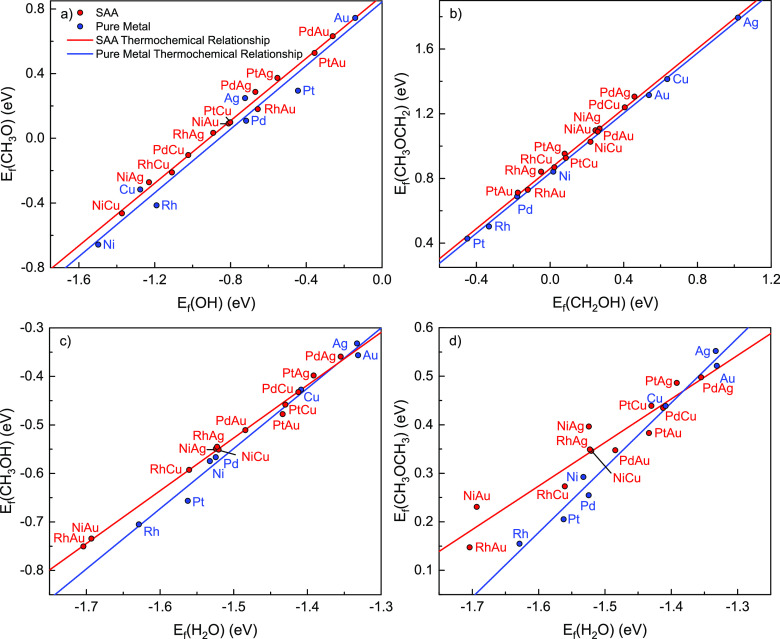
TCS relations of the formation energy *E*_f_ corresponding to fragments of (a) CH_3_O vs OH, (b) CH_3_OCH_2_ vs CH_2_OH, (c) CH_3_OH vs H_2_O, and (d) CH_3_OCH_3_ vs H_2_O bound in the most stable adsorption site on pure metals (blue) and SAAs (red). Adsorbates on SAAs were bound to their most stable dopant site. Corresponding linear regression fitting parameters and error analysis are given in Table S3.

The formation energies of the adsorbates on their most stable sites studied in this work are given in Table S2. The results indicate that the formation energies of closed-shell molecules do not change much after doping the Cu, Ag, and Au surfaces with transition metal atoms. According to the calculated formation energies, the open-shell fragments, including CH_3_O, OH, CH_3_OCH_2_, and CH_2_OH, show a greater range of variation which is around 1.4 eV (Figure 3a,b). On the other hand, the range of variations in the formation energies of H_2_O, CH_3_OH, and CH_3_OCH_3_ on different surfaces is less than 0.50 eV (Figure 3c,d). Different from open-shell fragments, these three adsorbates are closed-shell molecules that bind to the dopant top sites on most SAA surfaces via the O atoms. A possible reason for this trend is that adsorbates tend to form chemical bonds with the surface as the level of saturation reduces. In this situation, the covalent contribution will be more pronounced so that chemical bonds with transition metals generally exhibit greater strength compared to those with coinage metals.^58^

For the majority of the adsorbates, the formation energies on the SAA surfaces lie between those on their monometallic counterparts, which is in line with our previous results.^35^ In general, the formation energy of each adsorbate on the surface of the pure noble metal is much higher (indicating lower stability) than that on the pure transition metal (Table S2). This is also reflected in the binding of the adsorbates on SAAs, that is, stronger binding is observed on/around the dopant atom than on the surface of the host metal. However, we found that the above trend changes when the formation energies of species on noble and transition metals are close enough. For example, the adsorption energies of CH_3_O on Pt/Ag(111) and Pd/Ag(111) are higher than both of their monometallic counterparts as CH_3_O has similar formation energies on Pt(111), Pd(111), and Ag(111) (with energy differences lower than 0.15 eV) (Figure 3a). Another exception occurs in closed-shell species, including H_2_O, CH_3_OH, and CH_3_OCH_3_. For these three molecules, adsorbing on the dopant site of Ni/Au(111) and Rh/Au(111), which have the top two most positively charged dopant atoms (0.32 and 0.12 in units of *e*, respectively),^58^ results in higher stability compared to adsorbing on the corresponding pure transition metals (Figure 3c,d).

On pure metal surfaces, the adsorption energies of various molecular fragments scale linearly with those of other adsorbates with similar adsorption structures.^28−30^ In this study, the adsorption behavior of complex adsorbates on SAAs is investigated against that of simpler adsorbates (by substitution of CH_3_ by H). The TCS relations are applied to rationalize the relationship between formation energies of similar adsorbates, which can be of assistance in speculating on the formation of certain organic adsorbates, including CH_3_OH, CH_3_O, CH_3_OCH_3_, CH_3_OCH_2_, CH_2_OH, and CH_2_O and inorganic fragments H_2_O and OH.

Based on the similarity in the binding configuration, CH_3_O and OH were first paired up to investigate the relationship of their adsorption behaviors on pure metals and SAAs. TCS relations of the formation energies of these two adsorbates were then developed (Figure 3). The results of the formation energy indicate that there are strong linear relations of the formation energies of these two open-shell fragments on both pure metals and SAAs (pure metal *R*^2^ = 0.967, SAA *R*^2^ = 0.991). The formation energies of CH_3_OCH_2_ and CH_2_OH adsorbing on the top site also exhibit strong linear correlations (pure metal *R*^2^ = 0.999, SAA *R*^2^ = 0.990) (Table S3). The results indicate that for open-shell fragments, the formation energies exhibit a linear relation if they adsorb to the same site types. The Student *t*-test was then applied to verify if the slopes and intercepts of the two linear relationships of pure metals and SAAs are statistically different (Table S3). The result shows that the TCS relations for the OH and CH_3_O pair of adsorbates have the same slopes and intercepts in these two sets of materials at the 90% confidence level. For the relationship between CH_2_OH and CH_3_OH_2_, their intercepts in pure metals and SAAs are statistically the same at the 90% confidence level but become different at the 99% confidence level. Our results indicate that the linear relationship of the pure metals has the potential to provide good predictions of the adsorption strength of more complex adsorbates on SAA surfaces. A general observation from our calculations is that after the hydrogen atom is replaced with CH_3_, the formation energy of the resulting species exhibits a good linear correlation with the formation energy of the original adsorbate on both pure metals and SAAs.

Moving on to the closed-shell molecules, H_2_O, CH_3_OH, and CH_3_OCH_3_ bind to the dopant top site via their lone pairs. These fragments are Lewis bases as they are inclined to donate electronic density to the surface. In this study, the validation of TCS for the formation energies of H_2_O, CH_3_OH, and CH_3_OCH_3_ was examined (Figure 3c,d). The calculations show that the formation energies of CH_3_OH and those of H_2_O exhibit a strong linear relation (pure metal *R*^2^ = 0.986, SAA *R*^2^ = 0.995). Additionally, there is a slightly weaker linear relationship for the formation energies of CH_3_OCH_3_ and those of H_2_O (pure metal *R*^2^ = 0.983, SAA *R*^2^ = 0.912) (Table S3). These results are in line with our previous conclusion that closed-shell Lewis base fragments have formation energies that scale well with one another if they bind to the same site on the surface.

For the H_2_O and CH_3_OH adsorbate pair, the Student *t* test reveals that the two relations have the same intercepts at 99% confidence, while for the H_2_O and CH_3_OCH_3_ set, they have different intercepts and slopes at that confidence level (Table S3). Our results show that the TCS relations of CH_3_OH and CH_3_OCH_3_ for pure metals and SAAs have the same slopes and intercepts at the 90% confidence level. Thus, it appears appropriate to use a universal scaling relation for both pure metals and SAAs to make predictions about formation energies of CH_3_OH from H_2_O, as well as formation energies of CH_3_OCH_3_ from CH_3_OH, where only one of the H atoms in the adsorbate is replaced by a CH_3_ group. However, we cannot make predictions about the formation energies of CH_3_OCH_3_O directly from H_2_O via a universal scaling relation. This result indicates that for H_2_O, CH_3_OH, and CH_3_OCH_3_, the accuracy of predicting the SAAs’ TCS relationship using the pure metal TCS relationship decreases as the similarity of the adsorbate decreases.

On the other hand, the CH_2_O formation energies are not correlated with those of any of the above fragments due to the special chemical properties of this species and its unique bidentate adsorption geometries. A possible reason may be that the formation energies of density acceptors and those of density donors that bind to the same SAA surface site do not correlate. The TCS for donor adsorbates depends on the ability of each surface to accept electron density rather than the ability of the adsorbate to donate it; the surface-accepting properties remain constant irrespective of the adsorbate, and thus donor species formation energies are correlated with one another.^35,60^ As CH_2_O adsorbs on the surface via both one positively charged C atom and one negatively charged O atom, the adsorbate can both accept and donate density to the surface. Thus, it is no surprise that CH_2_O formation energies do not correlate with those of traditional Lewis bases.

### 3.2 Catalytically Relevant Bond Coupling/Dissociation Reactions

**Figure 4:**
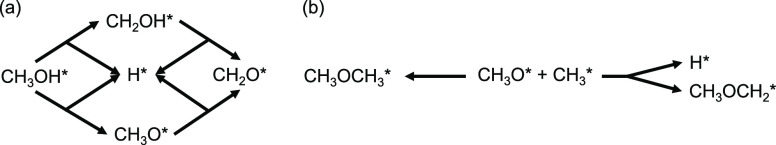
Two biomass reforming related reaction pathways for the (a) decomposition of CH_3_OH and (b) synthesis of CH_3_OCH_3_ on the different surfaces considered in this work.

We proceed further to calculate and compare the activation energies and reaction energies of several common elementary steps involving the oxygenated organic fragments discussed above on the surfaces of SAAs and pure metals. Among the wide range of possible elementary steps, we chose two reaction pathways, the initial decomposition of methanol and the synthesis of CH_3_OCH_3_ from CH_3_ and CH_3_O, in order to study the catalytic performance of SAAs on pertinent reactions in those fragments (Figure 4).

The elementary steps of methanol dissociation, including C–H activation in CH_3_OH and CH_3_O and O–H activation in CH_3_OH and CH_2_OH, were thus explored (Figure 4a). Further, C–O coupling was investigated for CH_3_OCH_3_ synthesis from CH_3_ and CH_3_O by focusing on the mechanism and the energy barriers on SAAs (Figure 4b). By comparing the DFT-computed energetics of these elementary steps on different catalyst surfaces, we found that some of the SAAs are predicted to exhibit lower activation energies, allowing decomposition reactions to occur readily. Thus, we investigate the catalysis of bond formation and scissions on SAAs to shed light on their mechanistic routes while comparing them to pure metals. In addition, we highlight a selection of systems that are promising for experimental/practical applications and build BEP relationships for our set of reactions on SAAs.

#### 3.2.1 C–H Activation in Methanol

**Figure 5:**
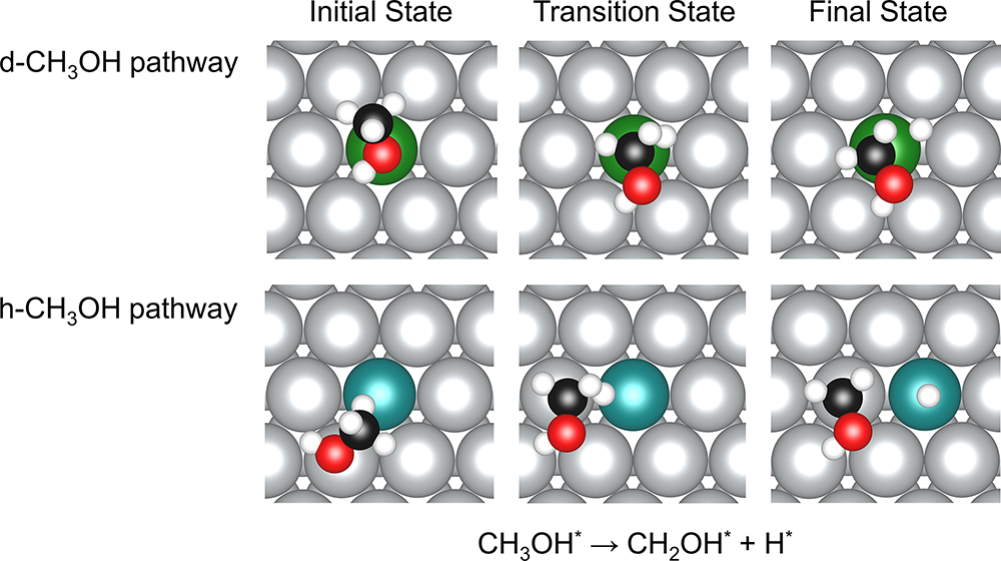
Initial states (left), transition states (middle), and final states (right) for C–H dissociation in CH_3_OH on Pd/Ag(111) and Pt/Ag(111) SAAs for the “d-CH_3_OH” (top) and “h-CH_3_OH” (bottom) dissociation pathways. A five-layer slab was used in these calculations along with a *p*(4 × 4) unit cell. Only a portion of the cell is shown for clarity.

The C–H bond dissociation in CH_3_OH was first investigated. From the results of CH_3_OH formation energies we discussed above in Section 3.1.1, CH_3_OH is more stable (adsorbs more strongly) on the dopant top sites of SAAs, except for Pd/Cu, Pt/Ag, and Pt/Cu where it prefers the nearest host top sites. In this study, two pathways of C–H activation are investigated for all SAAs initialized with the different adsorption configurations of CH_3_OH. Based on the position of the CH_3_OH adsorbates in the initial states, these two reaction pathways are named as “host-CH_3_OH (h-CH_3_OH)” and “dopant-CH_3_OH (d-CH_3_OH),” respectively (Figure 5). Our calculations show that the d-CH_3_OH pathway has lower-energy O–H dissociation transition states on all SAAs. In this study, we will mainly discuss the pathways with lower-energy transition states for each step on SAAs.

At the start of the dehydrogenation reaction, CH_3_OH is adsorbed at the dopant top site via the O atom. Then, one of the H atoms close to the surface is activated, and the C–H bond becomes elongated. As the carbon atom remains sp^3^-hybridized after the loss of the hydrogen atom, the adsorbate changes from adsorption through the O atom to adsorption through the C atom at the dopant top site, forming a “CH_2_OH-like” transition state. After that, the H atom detaches from the C atom and adsorbs on the dopant–host–host hollow site of the SAAs. By comparing the configurations of the transition states in the two pathways (h-CH_3_OH versus d-CH_3_OH), we deduce that the higher energy of the transition states of the less favorable pathway (d-CH_3_OH) may be caused by the less stable adsorption of CH_2_OH.

We then proceed to compare the energy barriers of the C–H activation between SAAs and pure metals. The C–H scission kinetic barriers on Ag(111), Au(111), and Cu(111) are 1.91, 1.52, and 1.40 eV, respectively, whereas on Ni(111), Pd(111), Pt(111), and Rh(111), these are 0.78, 0.64, 0.63, and 0.67 eV, respectively (Table S4). The result indicates that on transition metals, C–H activation occurs more readily compared to coinage metals, demonstrating that SAAs can reduce the reaction barriers of host metals.

According to our calculations, the activation energies of the host metals can be reduced by doping them with transition metal atoms. Significant reductions in the activation energies of C–H scission were thus observed after the addition of Rh atoms, with activation energies of 0.59, 0.70, and 0.69 eV for Rh/Ag(111), Rh/Au(111), and Rh/Cu(111) SAAs, respectively. These energy barriers are similar as those on pure Rh metal. Although not as good as Rh, Pt dopant atoms are also effective in reducing the energy barrier for C–H activation. For example, kinetic barriers on Pt/Ag(111), Pt/Au(111), and Pt/Cu(111) are 0.81, 0.78, and 0.88 eV, respectively. Ni-doped Ag(111), Au(111), and Cu(111) have values of 0.94, 1.06, and 0.88 eV, respectively (Table S4). These Ni-doped SAAs also exhibit low activation energies for the elementary steps, though higher than Rh- and Pt-doped SAAs. Pd possesses the least capacity to reduce the energy barrier of the reaction, whereas Pd/Ag(111), Pd/Au(111), and Pd/Cu(111) have C–H activation barriers of 1.10, 1.07, and 1.09 eV, respectively (Table S4). When comparing to the energy barriers of their pure host metal counterparts, we find that the energy barrier for this reaction drops most significantly (>0.8 eV) at the surface of Ag after the addition of dopant atoms. Furthermore, the activation energies drop by more than 0.5 and 0.3 eV compared to the energies on pure Au and Cu. Note that the activation energies mentioned in this section refer to d-CH_3_OH pathways as they have lower energies of transition states on all SAAs compared to their h-CH_3_OH pathways.

The lower-energy barriers in the SAAs compared with the pure metal hosts can be explained by the analysis of the formation energy discussed previously. After the addition of dopant atoms into pure host metals, the formation energy of both the initial and transition states of the reaction is decreased, but the degree of stabilization varies between the initial and final states. The decrease in the formation energy of the initial state is attributable to the fact that CH_3_OH is adsorbed on the top site of the dopant atom rather than on the host metal. As discussed earlier in Section 3.1.2, the formation energy of CH_3_OH, a closed-shell fragment, on individual metal surfaces does not vary much after doping with transition metals. On the other hand, inspection of the geometry of the transition state (H···CH_2_OH) of the d-CH_3_OH pathway shows that this configuration entails binding to the dopant via the C atom as a “final state-like” configuration. In agreement with our conclusions in Section 3.1.2, the adsorption energy of CH_2_OH drops dramatically when adsorbing to the dopant top site. In addition, the H adatom also exhibits a lower formation energy when binding to the dopant–host–host hollow site as demonstrated by Darby et al.^35^ In this case, a reasonable deduction is that after the addition of the doped atoms, the catalyst stabilizes the transition state more than the initial state, resulting in a lower activation energy, which is supported by the formation energies discussed in Section 3.1.2.

#### 3.2.2 O–H Activation in Methanol

**Figure 6:**
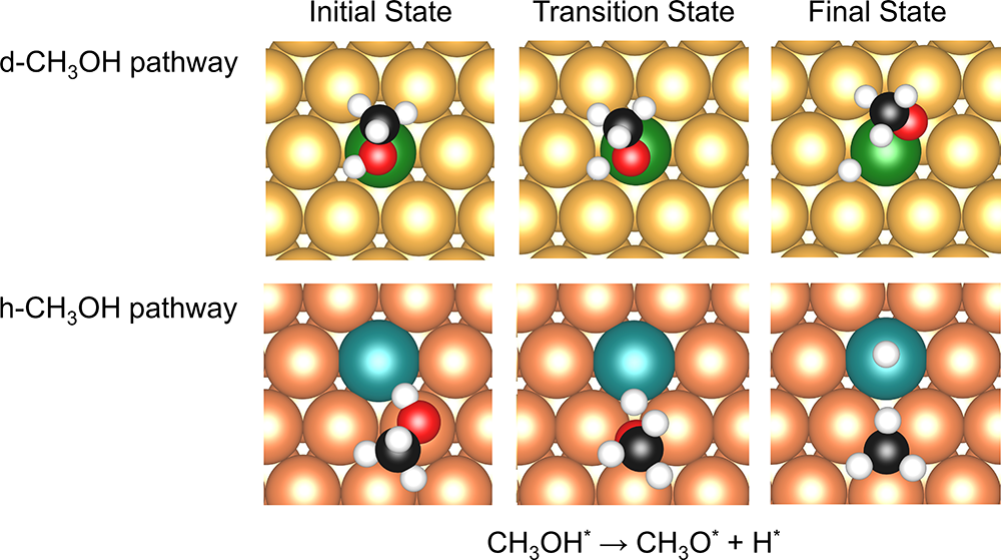
Initial states (left), transition states (middle), and final states (right) for O–H dissociation in CH_3_OH on Pd/Au(111) and Pt/Cu(111) SAAs for the “d-CH_3_OH” (top) and “h-CH_3_OH” (bottom) dissociation pathways. A five-layer slab was used in these calculations along with a *p*(4 × 4) unit cell. Only a portion of the cell is shown for clarity.

The same finding can also explain the different reaction pathways that involve the O−H activation elementary steps. Figure 6 shows the two CH_3_OH dissociation pathways, the transition states of which were labeled as “h-CH_3_OH” and “d-CH_3_OH” for CH_3_OH binding to host and dopant site in the initial states, respectively. As demonstrated by Darby et al.,^35^ d-CH_3_OH transition states have lower formation energy than h-CH_3_OH ones on Ni/Au(111), Ni/Ag(111), Ni/Cu(111), Pd/Au(111), Rh/Au(111), and Rh/Ag(111), while other SAAs favor the h-CH_3_OH pathway. Our calculations, which use a different computation setup, are in line with this result and provide further details for both pathways on all SAAs considered (Table S5). This trend suggests that a lower energy of the initial state may not always result in a lower energy of the transition state on each SAA, and it hints at a connection between the various reaction pathways and the formation energies of CH_3_O reported in Section 3.1.2.

More specifically, we discovered that, in the final state, CH_3_O always binds to the host site for the h-CH_3_OH pathways, while it binds to dopant sites for the d-CH_3_OH pathways. According to the formation energies of CH_3_O, this species prefers to adsorb on dopant metals on all of the SAAs that favor the d-CH_3_OH pathway, including Ni/Au(111), Ni/Ag(111), Ni/Cu(111), Pd/Au(111), Rh/Au(111), and Rh/Ag(111). As a result, lower energies are calculated for the transition states of this pathway, where CH_3_O lies close to dopant atoms, contrary to the h-CH_3_OH pathways. For other SAAs, except for Pt/Au(111), CH_3_O prefers to adsorb on the host metal instead of dopant sites, which is consistent with the structure of the methoxy-like transition and final states. One possible explanation for the exception of Pt/Au is that the H adatoms are more likely to adsorb on the dopant top sites than on the dopant–host–host hollow sites, thereby driving CH_3_O to the host site due to steric effects.^35^

#### 3.2.3 C–H Activation in Methoxy

**Figure 7:**
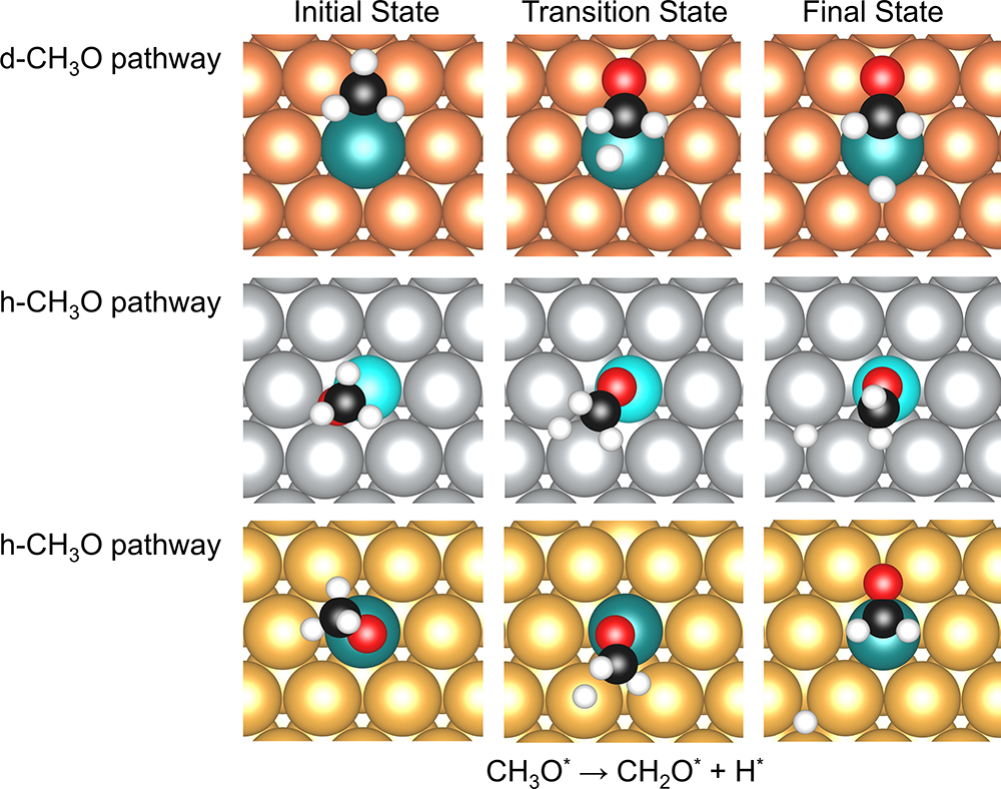
Initial states (left), transition states (middle), and final states (right) for C−H dissociation in CH_3_O on Pt/Cu(111), Ni/Ag(111), and Pt/Au(111) SAAs for the “d-CH_3_O” (top) and “h-CH_3_O” (middle and bottom) dissociation pathways. A five-layer slab was used in these calculations along with a *p*(4 × 4) unit cell. Only a portion of the cell is shown for clarity.

The cleavage of the C–H bond in CH_3_O bound to the dopant top and the dopant–host–host hollow sites on every SAA and pure metal surface was further examined. Similar to the C–H and O−H activations in CH_3_OH, different initial states lead to different pathways. If CH_3_O adsorbs to the dopant top site, C–H activation will occur on the nearest host site. On the other hand, if the CH_3_O fragment adsorbs to the dopant–host–host hollow site, the C–H activation will occur on either the dopant or the host site. In this case, it is difficult to use the initial state to define the reaction pathway; instead, based on where the activation happens, these two reaction pathways are named as “dopant-CH_3_O (d-CH_3_O)” and “host-CH_3_O (h-CH_3_O)” (Figure 7).

For most SAAs, d-CH_3_O pathways have lower formation energies of transition states compared to h-CH_3_O pathways. However, Pt/Au(111) and Rh/Au(111) are two notable exceptions (Figure 7). On these SAAs, the C–H cleavage of CH_3_O occurs only on the nearest host site. The CH_3_O formation energies indicate that for Pt/Au(111) and Rh/Au(111), the configurations are more stable (have lower energies) when CH_3_O adsorbs on the dopant top sites instead of the dopant–host–host hollow sites. As the C–O bond of CH_3_O is not perpendicular to the plane, these configurations allow the carbon atom to get closer to one of the nearest host atoms (Figure 7).

On the other hand, the CH_3_O molecules are located at the dopant–host–host hollow sites with their C–O bonds perpendicular to the surface at the beginning of the C–H activation on other SAAs. During the dissociation, the C atom moves close to the host atom, and the O atom translates from the hollow site to the host bridge site, causing the C–O bond to gradually tilt toward the plane. In this case, one of the H atoms close to the dopant atom moves away from the carbon atom, thereby breaking the C–H bond on top of the dopant. Subsequently, the H adatom relaxes to one of the dopant–host–host hollow sites, while CH_2_O adsorbs on the dopant–host–host hollow site on the other side of the dopant (Figure 7). Further, we found that CH_3_O binding on mixed hollow sites can also perform C–H activation on host atoms on Ni/Ag, Ni/Cu, Pd/Ag, Pd/Cu, Pt/Cu, and Rh/Cu SAAs when the carbon atom tilts toward the host atoms, which requires higher energy (Table S6). Unlike the C–H and O–H activation in CH_3_OH, the lowest energy CH_3_O C–H dissociation transition state always coincides with the lowest energy CH_3_OH adsorption pattern in initial states. In the following, we will mainly focus our discussions on the pathway with a lower transition state formation energy for each SAA.

On pure transition metals, Pt(111) has the lowest energy barrier, which is only 0.18 eV, followed by Pd(111), Rh(111), and Ni(111) having larger energy barriers of 0.57, 0.60, and 0.86 eV respectively. Pure coinage metals, including Ag and Cu, have higher activation energies than transition metals reaching 1.16 and 1.22 eV, respectively. An anomaly is that the reaction barrier of Au(111), which is 0.76 eV, is lower than that of the transition metal Ni. The energy barrier on Au is similar to that computed previously by Réocreux et al.^61^ The calculated formation energies provide a plausible explanation for this exception; thus, the lower activation energy on Au(111) may be due to the exceptionally high formation energy of CH_3_O on this surface, resulting in a higher initial state energy for the C–H activation (Table S6).

For SAAs, Ni-, Pd-, Pt-, and Rh-doped Ag(111) have C–H activation barriers of 0.74, 0.78, 0.45, and 0.42 eV, respectively, and those metals doped in Cu(111) have corresponding activation energies of 0.76, 1.00, 0.73, and 0.56 eV (Table S6). These data show that doping Ag(111) and Cu(111) with transition metal atoms significantly reduces activation energies. Previous calculations of the formation energy have shown that both the initial states and the transition states of C–H activation are affected by the addition of the dopant atoms. The resulting decrease in the activation energy is because dopant atoms have a stronger stabilizing effect on the transition state than that on the initial state. On the other hand, for Au-based SAAs, the activation energy of the reaction does not change significantly. There are two possible explanations for this trend. First, compared to Ag(111) and Cu(111), the C–H activation in CH_3_O on Au(111) exhibits a relatively low energy barrier, which is not only lower than that on Ni(111) but also close to that on Pt(111) and Rh(111) (0.16 and 0.19 eV higher than Pd(111) and Rh(111), respectively). The low activation energy can be attributed to the less stable initial state indicated by the high formation energy of CH_3_O on Au(111). Also, C-H activation occurs on the nearest gold atoms of the Pt/Au(111) and Rh/Au(111) SAAs, which weakens the interactions between adsorbates and dopant atoms, resulting in higher potential energies for the transition states.

#### 3.2.4 C–H Activation in Hydroxymethyl

**Figure 8:**
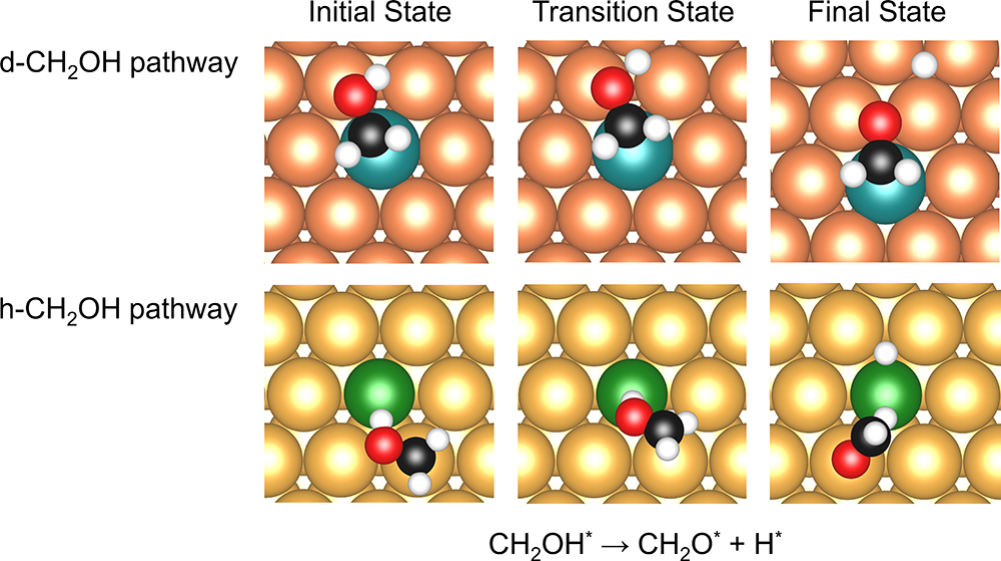
Initial states (left), transition states (middle), and final states (right) for O–H dissociation in CH_2_OH on Pt/Cu(111) and Pd/Au(111) SAAs for the “d-CH_2_OH” (top) and “h-CH_2_OH” (bottom) dissociation pathways. A five-layer slab was used in these calculations along with a *p*(4 × 4) unit cell. Only a portion of the cell is shown for clarity.

According to the previous discussion in Section 3.1.1, CH_2_OH prefers binding to the dopant top site for each SAA. However, similar to the O–H scission of CH_3_OH, although CH_2_OH may not adsorb on the most stable sites, the transition states can have lower energies, making the elementary step more facile. In this case, we investigated C–H activation in CH_2_OH, which occurs around the nearest host atoms and on the dopant atoms of SAAs for various initial state configurations. Two reaction pathways were investigated, which are referred to as the “host-CH_2_OH” (h-CH_2_OH) pathway and the “dopant-CH_2_OH” (d-CH_2_OH) pathway based on where the CH_2_OH binds to at the initial state configurations (Figure 8).

In the d-CH_2_OH pathway, CH_2_OH binding to the top site of the dopant atom via the C atom, such that the H and O atoms will extend beyond the dopant toward the host, is a prerequisite for enabling the O–H scission to take place on the host atom. Subsequently, as the H atom moves farther away from the CH_2_OH adsorbate, the O–H bond breaks at the host site. After the breaking of the O–H bond, the hydrogen adatom diffuses into the host hollow site, and CH_2_O binds on the dopant top site (Figure 8).

On the other hand, the CH_2_OH species undergoes O–H scission via the h-CH_2_OH pathway when the fragment adsorbs to the top site of the host atom, with the O–H bond parallel to the SAA surface. Along the h-CH_2_OH pathway, the molecular plane of CH_2_OH is slowly rotated until it is perpendicular to the surface of the metal. In the transition state, the H atom gradually approaches the dopant atom, and the fragment adsorbs on top of the SAA dopant atom through the H atom, where the O–H activation occurs. After cleavage, the H atom binds to the dopant site, while the O atom of CH_2_O binds to the top site of the host (Figure 8).

**Figure 9:**
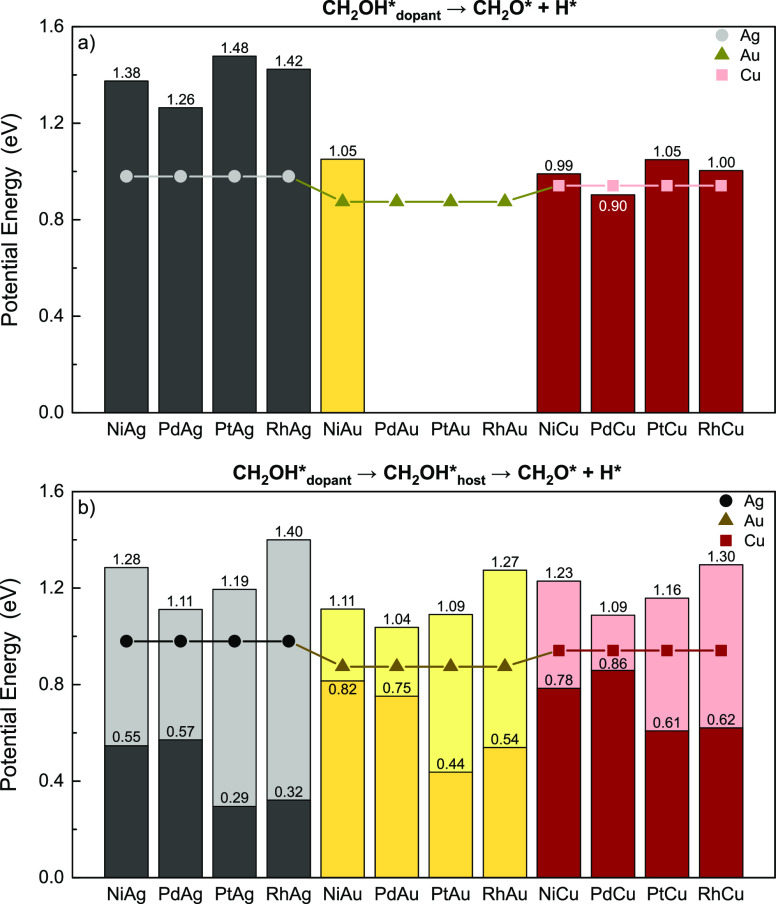
Energy barriers for (a) d-CH_2_OH and (b) h-CH_2_OH reaction pathways. The dark gray, dark yellow, and brown bars show the activation energy on SAAs, while the light gray, yellow, and pink bars represent the energy required for moving CH_2_OH binding to dopant top sites to host sites. The numbers on the top of the light gray, yellow, and pink bars represent the apparent activation energies on SAAs. The circles, triangles, and squares represent the activation energy on Ag(111), Au(111), and Cu(111), respectively. Three datapoints are missing as we were not able to find a transition state for the corresponding pathway for Pd/Au, Pt/Au, and Rh/Au. Reaction energies of these pathways can be found in Table S7.

The pathways of CH_2_OH dissociation on pure metals are similar to the d-pathway on SAAs. Among them, transition metals exhibit lower O–H scission barriers for the CH_2_OH species than pure host metals. In particular, Ni(111), Pd(111), Pt(111), and Rh(111) have barriers of 0.65, 0.72, 0.64, and 0.71 eV, while the barriers for Au(111), Ag(111), and Cu(111) are 0.98, 0.87, and 0.94 eV respectively. According to our calculations, on Ni/Au, Ni/Cu, Pd/Cu, Pt/Cu, and Rh/Cu, the O–H scission proceeds more favorably via the d-CH_2_OH pathway, having corresponding kinetic barriers of 1.05, 0.99, 0.90, 1.05, and 1.00 eV, respectively (Figure 9). On the other hand, the other SAAs favor the h-CH_2_OH pathway. The apparent activation energies on Ag-based SAAs [defined with the most stable initial state; see Section 2.2 of Computational Details; 1.28 eV on Ni/Ag(111), 1.11 eV on Pd/Ag(111), 1.19 eV on Pt/Ag(111), and 1.40 eV on Rh/Ag(111)] are higher than those on Au-based SAAs [1.04 eV on Pd/Au(111), 1.09 eV on Pt/Au(111), and 1.27 eV on Rh/Au(111)] when doping with the same transition metal atoms (Figure 9b).

According to the results of Section 3.1.1, CH_2_OH is more stable on dopant top sites such that the d-CH_2_OH pathway can occur directly with the O–H scission taking place on the host metal. As the reaction proceeds on the dopant atom, even though the formation energies of the initial states are drastically decreased by the dopant transition metal atoms, the transition states, where adsorbates are bound to mixed dopant-host sites, are less influenced. In this case, the activation energies on SAAs should be higher than those on pure metals, which is in line with the calculation result except for Pd/Cu(111) (Figure 9a).

On the other hand, the apparent activation energies of d-CH_2_OH pathway mentioned in this section can be divided into two parts—the energy barrier of CH_2_OH diffusion from dopant sites to host sites as well as the activation energy of C–H activation on dopant sites. Different from other dehydrogenation reactions studied here, the initial state plays an important role in this event as the energy difference between CH_2_OH on the dopant site and CH_2_OH on the host site is quite large (ranging from 0.23 to 1.08 eV) (Figure 9b). This could be due to insufficient charge mixing between the transition metal and the noble metal, causing the isolated dopant top site to exhibit a different reactivity compared to shared dopant–host–host hollow sites.^11,62,63^

Our calculations also show that the actual activation energies of C–H activation on dopant sites can be quite small on some SAAs, though a large portion of the energy barriers pertains to the diffusion of CH_2_OH. For instance, migration of the CH_2_OH fragment from dopant to host sites on Ni/Ag(111), Pt/Ag(111), Rh/Ag(111), Pt/Au(111), Rh/Au(111), and Rh/Cu(111) requires at least 0.73, 0.90, 1.08, 0.54, 0.73, and 0.68 eV, respectively, which account for more than 50% of the overall energy barriers (Figure 9b). This feature can be attributed to the unique configuration of SAAs. According to the d-CH_2_OH pathway, the dopant atoms greatly stabilize the transition states as C–H scission happens on the dopant atom; however, the initial states are less affected by the dopant atom due to their more “dopant–host mixed” adsorption patterns, although there is an energy penalty to form the patterns. The h-CH_2_OH and d-CH_2_OH reaction pathways indicate that SAAs have the potential to affect both the initial and transition states, and the reduction of the energy barrier depends on the differences in the ability to stabilize each of the two states.

#### 3.2.5 C–O Coupling between CH_3_O and CH_3_

It has been widely reported that SAAs exhibit significantly better activity and selectivity compared to “traditional” pure metal catalysts.^8^ In this context, the synthesis of CH_3_OCH_3_, an important chemical with uses as fuel, refrigerant, or propellant in aerosol products, could potentially benefit the use of SAA catalysts. We therefore studied the catalytic effects of SAAs with different bimetallic combinations [Ni-, Pd-, Pt-, and Rh-doped Cu(111), Ag(111), and Au(111)] for the C–O coupling steps between CH_3_O and CH_3_.

**Figure 10:**
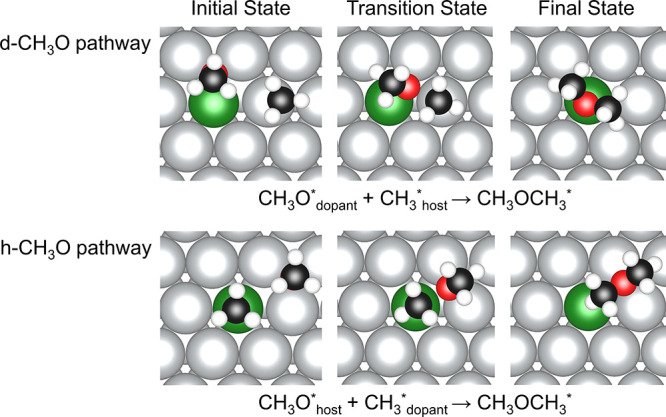
Initial states (left), transition states (middle), and final states (right) for C–O coupling between CH_3_ and CH_3_O on Ni/Ag(111) (Group 1 SAA) for the “d-CH_3_O” (top) and “h-CH_3_O” (bottom) coupling pathways. A five-layer slab was used in these calculations along with a *p*(4 × 4) unit cell. Only a portion of the cell is shown for clarity. Activation energies and reaction energies for group 1 SAAs and pure metals can be found in Table S8.

In order to investigate the role of dopant atoms in catalyzing C–O coupling, we built two different initial states for each SAA. For each of the two initial states, one of the two reactant adsorbates, CH_3_ or CH_3_O, was set on its most favorable dopant site, while the other was set on the nearest most favorable host site. Based on the position of CH_3_O, these two initial states were named as “dopant-CH_3_O (d-CH_3_O)” geometry and “host-CH_3_O (h-CH_3_O)” geometry (Figure 10). The formation energies of the d- and h-geometries on each SAA were first compared, revealing that for most of the SAAs, except for Ni/Au(111) and Rh/Au(111), the h-CH_3_O geometry is more stable. The plausible explanation is that for both Ni/Au and Rh/Au, the dopant atom of the surface gathers more positive charge compared to the other SAAs. Due to the strong electronegative character of the oxygen atom in CH_3_O, a more stable ionic-type bond is formed when CH_3_O adsorbs on the surface of the dopant atom in these two kinds of SAAs, resulting in more stable d-CH_3_O geometries.^58^

**Figure 11:**
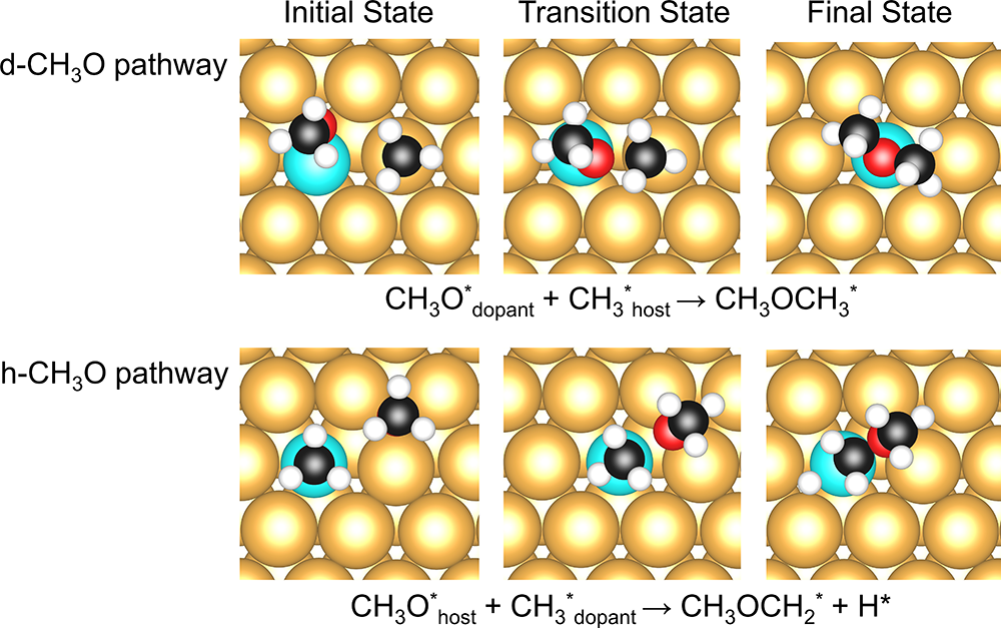
Initial states (left), transition states (middle), and final states (right) for C–O coupling between CH_3_ and CH_3_O on Ni/Au(111) (group 2 SAA) for the “d-CH_3_O” (top) and “h-CH_3_O” (bottom) coupling pathways. A five-layer slab was used in these calculations along with a *p*(4 × 4) unit cell. Only a portion of the cell is shown for clarity. Activation energies and reaction energies for group 2 SAAs and pure metals can be found in Table S9.

The C–O coupling steps that start from the d-CH_3_O and h-CH_3_O geometries were defined as the d-CH_3_O and h-CH_3_O pathways, respectively. Our calculations show that CH_3_ and CH_3_O can react with each other via two distinct elementary steps, producing either adsorbed CH_3_OCH_3_ or CH_3_OCH_2_ and an H adatom depending on the initial state and the SAA on which the reaction takes place. Thus, all SAAs can be divided into two groups; dopant atoms in group 1 SAAs, which include Pd/Au, Pd/Ag, Pd/Cu, and Pt/Cu, produce CH_3_OCH_3_ from both host-based and dopant-based initial states (Figure 10). For group 2 SAAs, which include Ni/Au, Rh/Au, Pt/Au, Pt/Ag, Rh/Ag, Ni/Ag, Rh/Cu, and Ni/Cu, the dopant atoms have the potential to activate the C–H bonds in CH_3_ along the h-pathway to produce CH_3_OCH_2_ and H adatoms (Figure 11). Similar to group 1 SAAs, pure Au, Ag, Cu, and Pd cannot activate the C–H bond of CH_3_ during the C–O coupling steps. However, Pt, Rh, and Ni have the potential to cleave the C–H bond to produce CH_3_OCH_2_ and a H adatom. In this case, the seven pure metals investigated in this research can also be divided into two groups. Group 1 includes three coinage metals (Cu, Ag, and Au) and Pd, while group 2 includes pure Ni, Pt, and Rh.

Our results show that for group 1 SAAs, whether CH_3_OCH_3_ can be generated on the alloy surface is independent of its initial geometry (Figure 10). At the beginning of the reaction, CH_3_O moves toward CH_3_. As the O atom approaches CH_3_, the C–O bond in CH_3_O is no longer perpendicular to the Pd/Ag(111) surface. In the case of the h-CH_3_O pathway, C–O coupling first occurs at the mixed hollow site to produce CH_3_OCH_3_, which subsequently diffuses to the host bridge site. For the d-CH_3_O pathway, the C–O coupling occurs at the dopant–host bridge site on Ni/Au(111), Pt/Au(111), and Rh/Au(111), whereas for other SAAs, this coupling happens at the mixed hollow site (Figures 10 and 11).

For group 2 SAAs, concerted C–H cleavage can occur during the C–O coupling elementary step from the initial state of the h-CH_3_O geometry (Figure 11). More specifically, dopant atoms have the potential to break the C–H bond of CH_3_ during this elementary step. On the contrary, the d-CH_3_O pathway for group 2 SAAs can only produce CH_3_OCH_3_ adsorbates on the surface as the dopant atoms in group 2 SAAs cannot break the C–H bond for CH_3_O.

One possible reason for these different chemical behaviors is that because of the proximity of the H for CH_3_ with the surface in the coupling transition states, some elementary steps involve concerted C–H activation (Figure 11). The C sp^3^ atoms strongly bind to the dopant top site on these group 2 SAAs, making it difficult for C atoms to detach from the surface. In this case, carbon atoms more readily bond with surface transition metal atoms rather than hydrogen. In addition, dopant–host–host fcc-hollow sites on group 2 SAAs have the potential to bond H atoms more strongly than group 1 SAAs, allowing the C–H bond in CH_3_ to break first. In this case, dopant atoms in group 2 SAAs can provide a reaction pathway with a lower-energy barrier for the C–O coupling by activating the H atoms in CH_3_ and keeping C atoms bound to the pure dopant site during the elementary step.^35^ By comparing the transition states of each SAA, we found that the distances between the C atom of CH_3_ and the dopant atom on the slab for group 1 SAAs are larger than those for group 2 SAAs (Table S13).

**Figure 12:**
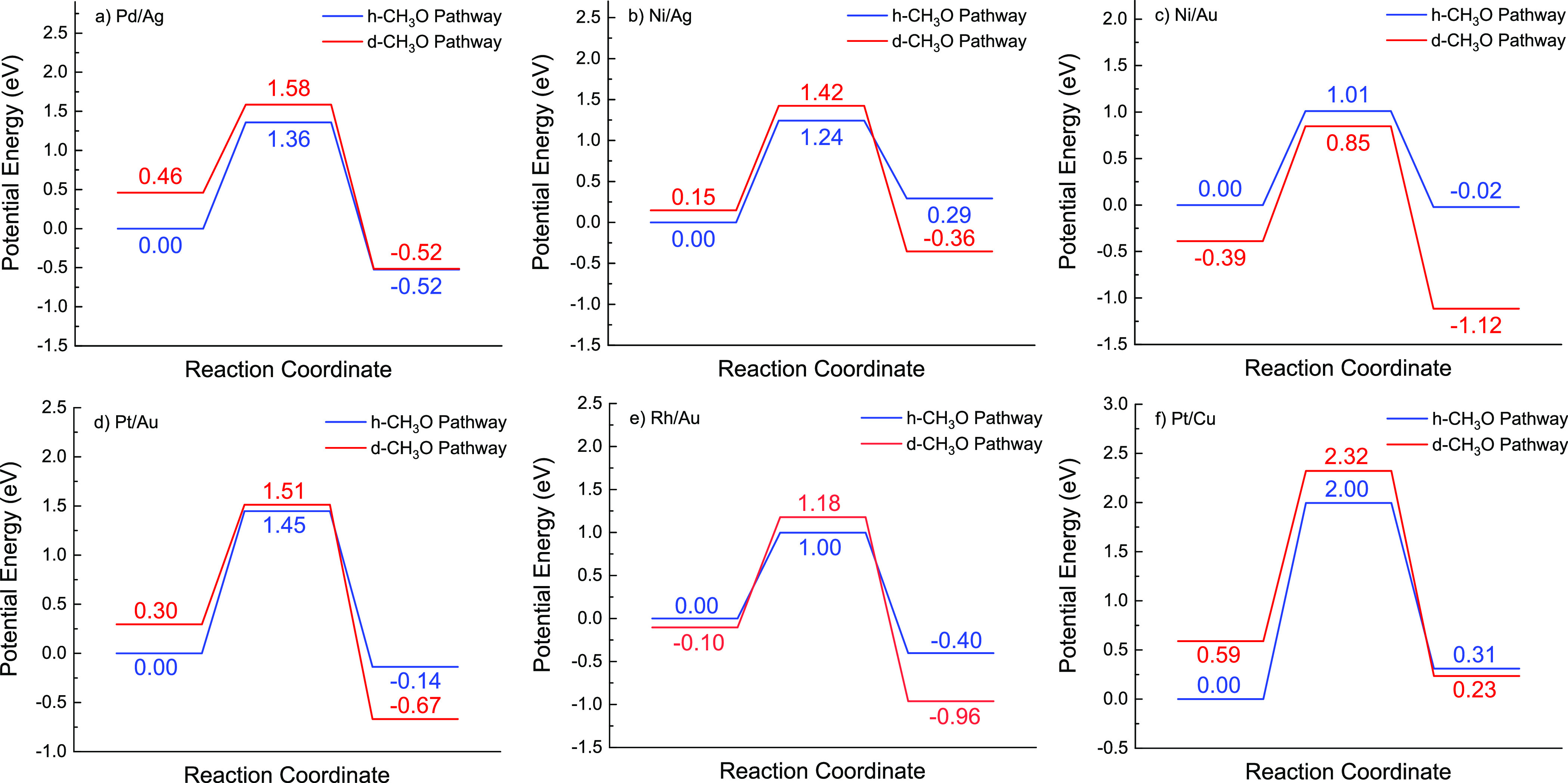
Energy profiles of d-CH_3_O and h-CH_3_O pathways for C–O coupling on (a) group 1 SAA Pd/Ag, (b) group 2 SAAs Ni/Ag(111), (c) Ni/Au(111), (d) Pt/Au(111), (e) Rh/Au(111), and (e) Pt/Cu(111).

For group 1 SAAs, the h-CH_3_O pathway has lower formation energies of both initial and transition states compared to the d-CH_3_O pathway, indicating that the h-CH_3_O pathway is more likely to occur on group 1 SAAs (Figure 12a,f). Similarly, comparing the formation energy of transition states of group 2 SAAs shows that h-CH_3_O pathways have a lower transition state energy on all SAAs investigated except for Ni/Au(111) (Figure 12b,c,d,e). The energy profiles of the h- and d-CH_3_O pathways on Ni/Ag(111) and Ni/Au(111) are further compared (Figure 12b,c). As CH_3_O in the transition state remains adsorbed around the dopant atom for the d-CH_3_O pathway, the anomalous behavior of Ni/Au(111) (Figure 12c) may also result from the high positive charge accumulated at the dopant atom leading to a stronger binding of CH_3_O thereon and the stabilization of the transition state.

### 3.3 Comparing Catalytic Activities between SAAs and Pure Metals

To highlight the potential benefits of the SAAs for the chemistries of interest, we herein perform comparisons of catalytic activities between SAAs and pure metals for the dehydrogenation and C–O coupling steps. To this end, we calculate the transition state formation energy differences between SAAs and their respective pure host and dopant metals. Note that although pure dopant metal surfaces have lower apparent activation energy for dehydrogenation compared to SAAs, they suffer from poor selectivity and severe poisoning issues (coke formation).^64,65^ Hence, in this section, we mainly focus on comparing the catalytic performance between SAAs and their pure host metal counterparts. Additionally, apparent activation energy differences among SAAs are compared in the Supporting Information.

#### 3.3.1 C–H and O–H Bond Scission Reactions

**Figure 13:**
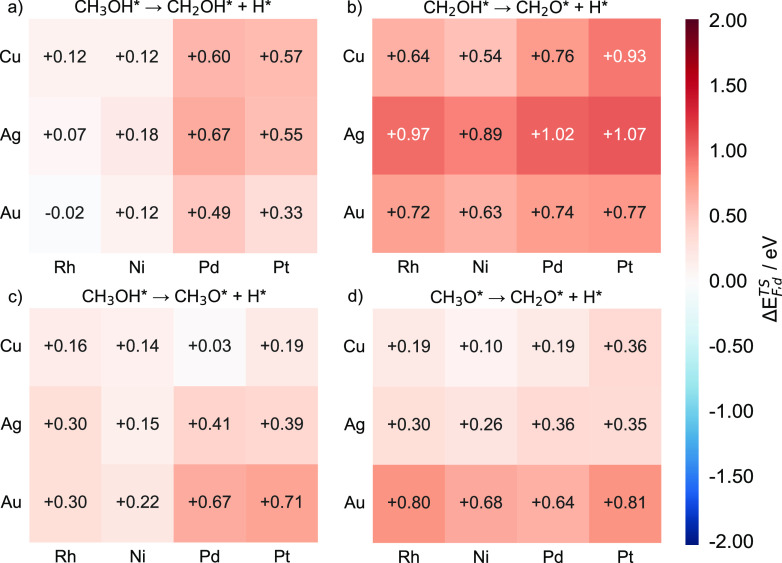
Heatmap charts of the transition state formation energy differences (Δ*E*_F,d_^TS^) for the following dehydrogenations: (a) CH_3_OH C–H activation, (b) CH_2_OH O–H activation, (c) CH_3_OH O–H activation, and (d) CH_3_O C–H activation for Cu-, Ag-, and Au-based SAAs. Each SAA Δ*E*_F,d_^TS^ value is calculated as the formation energy of the lower-energy transition state (between the d- and h-pathways) minus the energy of the corresponding pure dopant metal.

Although most SAAs have a lower formation energy of transition states than their monometallic hosts, their activities are also limited by the fact that host atoms (which exhibit higher activation energies) constitute the majority of sites on the catalytic surface (Figure 13). This is especially true for CH_2_OH O–H activation: using SAAs instead of their pure dopant metals as catalysts would increase the transition state formation energy by 0.54–1.07 eV (Figure 13b). Regarding CH_3_OH C–H activation, the activity of Pd(111) and Pt(111) would reduce since the activation energy would increase by 0.33–0.67 eV after forming SAAs with coinage metals (Figure 13a).

Transition state formation energies for CH_3_OH O–H activation and CH_3_O C–H activation are also higher on Au-based SAAs compared to the respective pure dopant atoms (Figure 13c,d). On the other hand, Rh/Au(111) and Pd/Cu(111) on CH_3_OH C–H and O–H scission, respectively, have similar formation energies as their pure dopant atoms. This result indicates that Rh and Pd single atoms doped on Au and Cu, respectively, can maximize their ability to stabilize the transition state. It has to be noted, however, that very low H abstraction barriers, such as those observed in pure dopants, lead to risks of overdehydrogenation and poisoning of the catalytic surface, as also demonstrated in our previous work on Pt/Cu(111) and Rh/Cu(111).^64,65^ Alloying such highly active metals with more inert hosts prevents the risk of poisoning.

**Figure 14:**
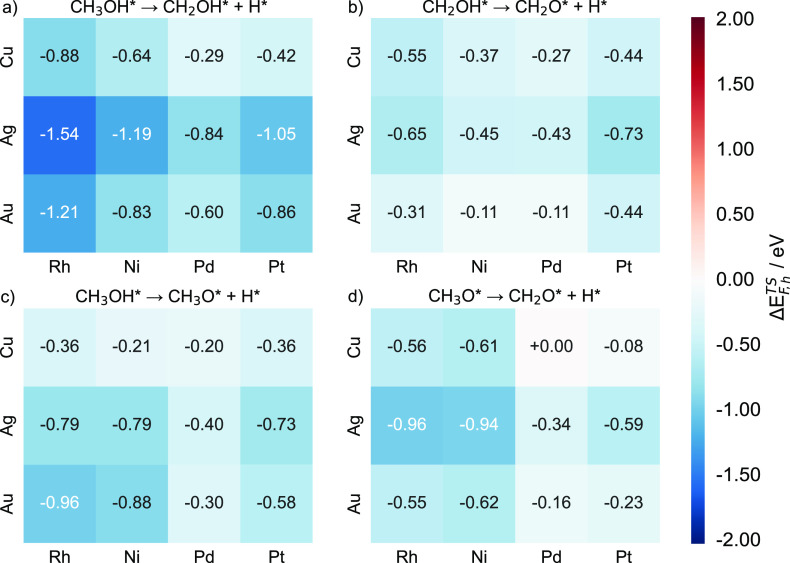
Heatmap charts of the transition state formation energy differences (Δ*E*_F,h_^TS^) for the following dehydrogenations: (a) CH_3_OH C–H activation, (b) CH_2_OH O–H activation, (c) CH_3_OH O–H activation, and (d) CH_3_O C–H activation for Cu-, Ag-, and Au-based SAAs. Each SAA Δ*E*_F_^TS^ value is calculated as the formation energy of the lower-energy transition state (between the d- and h-pathways) minus the energy of the corresponding pure host metal.

Furthermore, the transition state formation energy differences between SAAs and pure host metals (Δ*E*_F,h_^TS^) are calculated in order to compare the effects of coinage metal doping on reducing the activation energies (Figure 14). A negative sign for Δ*E*_F,h_^TS^ implies that the SAA exhibits lower transition state energy compared with the pure host metals, while a positive sign indicates the opposite. Some activation energies on pure metals can be quite low because of the high formation energies of the corresponding initial states (see Section 3.2.4). In this case, the elementary steps are hindered by the difficulty in adsorbing on pure metals, and the apparent activation energy (see Section 2.2 Energy Definitions) may be misleading. However, the formation energies of transition states can be seen as the apparent activation energy of an elementary step from a reference state that includes only gas-phase reference species and clean surfaces. Therefore, by using these formation energies, we can avoid the impact of initial state energy differences when comparing the activities of the SAA catalysts of interest.

Thus, by analyzing the values of Δ*E*_F_^TS^, we find that the apparent energy barrier for the CH_3_OH C–H activation drops most significantly on Ag(111) (absolute value of the decrease > 0.8 eV) after the addition of dopant atoms, followed by Au. On the other hand, doping of transition metal atoms has the least effect on increasing the activity of Cu(111) (Figure 14a). If we take into account the intrinsic activity of the host metals, we find that doping with transition metal atoms has a more pronounced effect on host metals, with originally poorer catalytic performance for CH_3_OH C–H activation. In addition, doping with Rh results in the highest reduction of the apparent activation energy, followed by doping with Pt and Ni. Nevertheless, doping with Pd atoms is predicted to have the smallest effect on reducing the energy barriers for CH_3_OH C–H activation.

Moving on to the dehydrogenation of CH_2_OH, Ag(111) is also the most suitable host for doping with transition metals, followed by Cu and Au. Additionally, doping Au(111) with Ni and Pd can only slightly change its apparent activation energies (Figure 14b). For the other two dehydrogenation steps, the addition of Pd is predicted to always have a lower impact on activation energies, especially for CH_3_O C-H activation on Pd/Cu(111), whose apparent activation energy barely changes (Figure 14c,d).

Our results show that the degree of reduction in the activation energy varies greatly (Figure 14). For example, doping Ag(111) with Rh is predicted to reduce the apparent barrier of CH_3_OH C–H activation by 1.54 eV, while doping Cu(111) with Pd would result in a barrier reduction of only 0.29 eV (Figure 14a). For CH_2_OH O–H activation, by doping Ag(111) with Pt, the transition state formation energy would decrease by 0.73 eV, which is nearly a 7-fold higher effect than that of doping Au(111) with Ni and Pd. These calculations demonstrate that by doping host metals with a minute amount of carefully chosen transition metals, the SAAs formed may exhibit significantly enhanced activities compared to the pure hosts.

#### 3.3.2 C–O Coupling between CH_3_O and CH_3_

**Figure 15:**
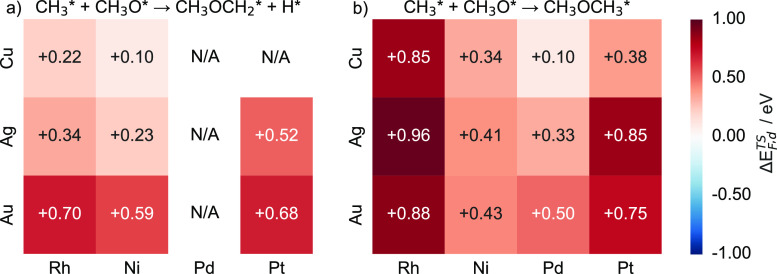
Heatmap charts of the transition state formation energy differences (Δ*E*_F,d_^TS^) for (a) CH_3_OCH_2_ and (b) CH_3_OCH_3_ intermediate formation for Cu-, Ag-, and Au-based SAAs. Each SAA Δ*E*_F,d_^TS^ value is calculated as the formation energy of the transition state minus the energy of the corresponding pure dopant metal.

Continuing the analysis with the C–O coupling step between CH_3_O and CH_3_, our calculations indicate that SAAs exhibit transition formation energies higher than those of their dopant counterparts (Figure 15). While C–O bonding is thus more readily performed on pure dopant metals, it is important to note that these transition metals may not be ideal catalysts for C–O coupling reactions: first, Ni, Pt, and Rh will perform the concerted step that involves C–H activation in CH_3_ during C–O coupling. In addition, pure dopant atoms exhibit lower activation barriers toward C–H scission in CH_3_ and CH_3_O, thereby reducing the yield of CH_3_OCH_3_.^35,64,65^

**Figure 16:**
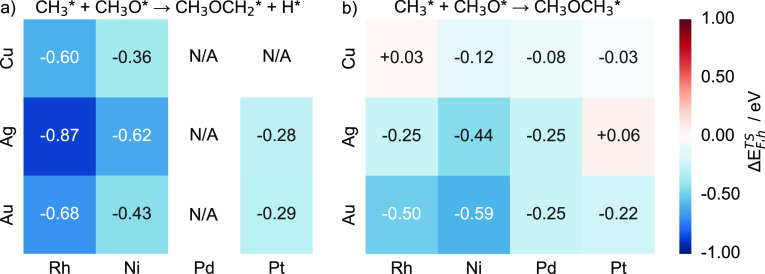
Heatmap charts of the transition state formation energy differences (Δ*E*_F,h_^TS^) for (a) CH_3_OCH_2_ and (b) CH_3_OCH_3_ intermediate formation for Cu-, Ag-, and Au-based SAAs. Each SAA Δ*E*_F,h_^TS^ value is calculated as the formation energy of the transition state minus the energy of the corresponding pure host metal.

Compared to pure hosts, most SAAs have a lower transition state formation energy for C–O coupling, indicating that doping transition metal atoms into coinage metal hosts can stabilize the transition states, thereby reducing their apparent activation energies except for Pt/Ag(111) and Rh/Cu(111) (Figure 16). The results indicate that introducing Pd into Au(111) and Ag(111) is more effective compared with doping it to Cu(111) (Figure 16b). On the other hand, doping with transition metal atoms has a more pronounced effect on reducing the apparent activation energies on the CH_3_OCH_2_ forming step, except for Ni/Au(111). In conclusion, Pd may be one of the suitable dopant atoms to produce SAAs for catalyzing CH_3_OCH_3_ synthesis.

### 3.4 BEP Relationships

**Figure 17:**
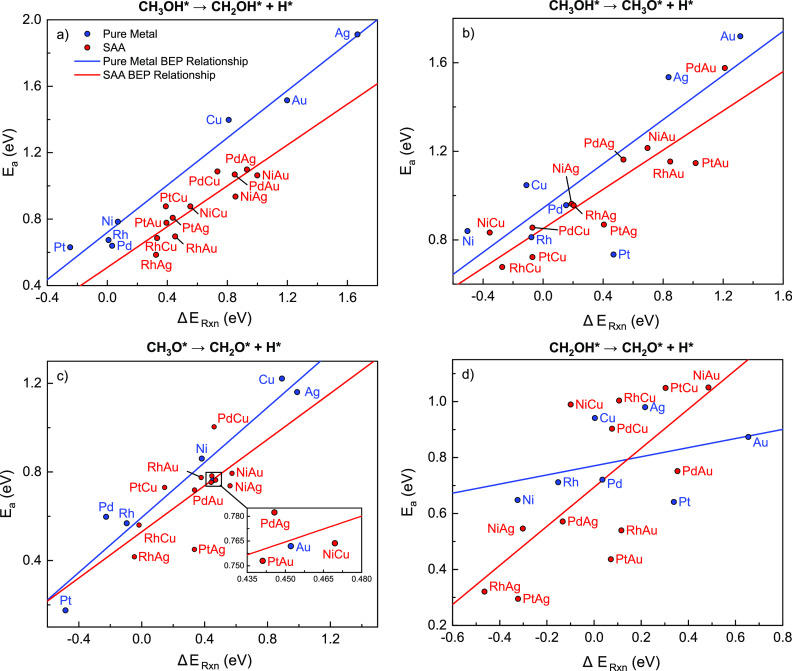
BEP relationships for (a) C–H activation in CH_3_OH, (b) O–H activation in CH_3_OH, (c) C–H activation in CH_3_O, and (d) O–H activation in CH_2_OH on pure metal(111) surfaces (blue) and SAA(111) surfaces (red). The plots show the activation energy as a function of the reaction energy. Linear fits are drawn for each reaction on each surface type. Corresponding linear regression fitting parameters and error analysis are given in Table S11.

The BEP relationship is a linear correlation between the activation energy and the reaction energy, which can be used to predict the performance of catalytic materials.^30−34^ BEP relationships for the CH_3_OH dissociation network (cleavage elementary steps) were first developed and compared with each other (Figure 17).

For pure metals (blue points/lines in Figure 17), the linear correlations capture well the relationship between the activation energy and the reaction energy for the C–H scission in CH_3_OH and the C–H activation in CH_3_O. In particular, the coefficients of determination, *R*^2^, and the mean average errors (MAEs) in the prediction of energy barriers from linear fits are as follows: *R*^2^ = 0.979 and MAE = 0.066 eV for C–H scission in CH_3_OH and *R*^2^ = 0.928 and MAE = 0.080 eV for H abstraction from CH_3_O (Figure 17a,c). On the other hand, poor BEP correlations are found for pure metals for the O–H activation step in CH_3_OH (MAE = 0.090 eV, *R*^2^ = 0.658), which can be attributed to the high reaction energy exhibited by outlier Pt(111) (Figure 17b). This anomaly may be caused by weaker binding of CH_3_O on Pt(111) as this species exhibits the second highest formation energy on Pt(111) among the pure metals (Table S2).

Moving on to the BEP linear regressions on SAAs (red points/lines in Figure 17), we find that C–H activation in CH_3_OH and O–H activation in CH_3_OH have low MAEs (0.07 and 0.10 eV, respectively) in the prediction of the activation energy from reaction energies, with relatively high *R*^2^ values of 0.756 and 0.780, respectively (Figure 17a,b), which is in contrast to the poor linear fit for C–H scission in CH_3_O (*R*^2^ = 0.452, MAE = 0.080 eV).

Interestingly, across the range of reaction energies considered here, the SAA BEP lines lie below those of the pure metals in all cases. In general, this indicates that SAAs exhibit broadly lower activation energies in comparison to those of the pure metal surfaces. Thus, SAAs combine both weak binding and low activation energy, allowing for improved catalytic performance. The Student *t*-test was then applied to detect if the BEP relationships between SAAs and pure metals are different from one another for each elementary step. Slopes and intercepts of pure metal and SAA BEP relations of three dehydrogenation steps mentioned in the last paragraph are not different at the 90% confidence level except for the intercept in CH_3_OH C–H activation. Thus, in the cases of CH_3_OH O–H scission and CH_3_O C–H scission, it appears appropriate to use a universal scaling relation for both pure metals and SAAs to make predictions about their catalytic performance, though in the case of CH_3_OH C–H scission, a specific BEP relationship for each type of material must be used (Table S12).

**Figure 18:**
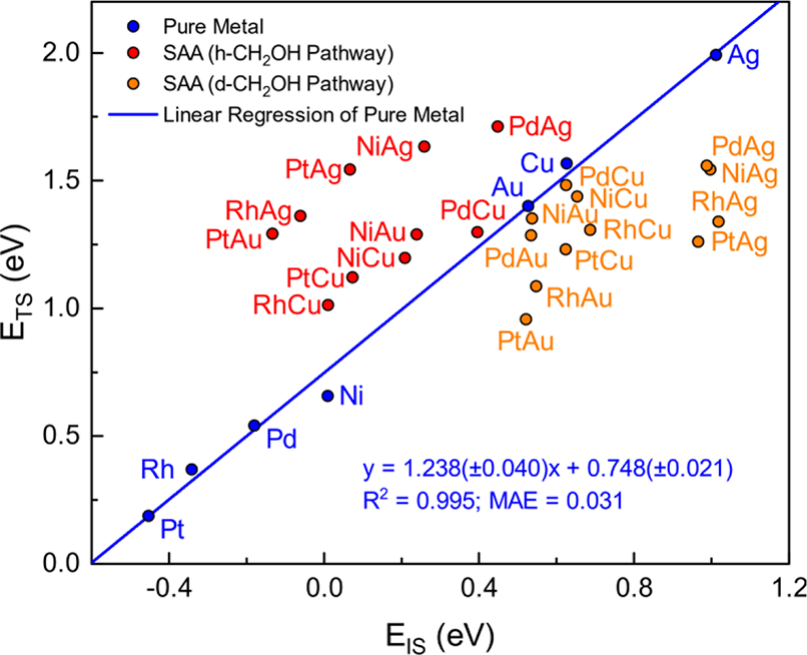
Energies of transition versus initial states for the O–H activation in CH_2_OH. A linear fit is drawn only for pure metals (blue). Red and orange circles indicate h-CH_2_OH and d-CH_2_OH pathways on SAAs, respectively. Formation energies can be found in Table S10.

While the aforementioned reactions exhibit strong BEP correlations, the O–H activation in CH_2_OH is completely different in this perspective (Figure 17d). The points are largely scattered, and no clear BEP linear relations are found for either SAAs or pure metals (*R*^2^ = 0.497, MAE = 0.024 eV for pure metals and *R*^2^ = 0.141, MAE = 0.099 eV for SAAs). However, a strong linear correlation between initial and final state energies is discovered with *R*^2^ = 0.995 (Figure 18). García-Muelas et al.^57^ also found that the O–H activation has a poor BEP relationship on pure metals, but the linear relation improves significantly if the potential energy of the initial states instead of the reaction energy was used as a descriptor.

As shown in Figure 18, the formation energies of CH_2_OH adsorbed on the host atoms of SAAs are quite similar to those on their corresponding pure host metals (the orange dots of the SAAs lie directly below the blue dots of the pure metals). In addition, it is revealed that the dopant atoms have the capacity to stabilize the transition states in the d-CH_2_OH pathway, with a stabilization strength following the trend: Pt > Rh > Ni > Pd (an exception is Ni/Au(111), which has a higher transition state energy than Pd/Au(111)) (Figure 18). Besides, when CH_2_OH binds to dopant atoms, the pertinent initial state energies are significantly lower compared to those of pure host metals. Interestingly, only the Cu-based SAAs and Ni/Au(111) have lower transition state formation energies in the h-CH_2_OH pathway compared to the d-CH_2_OH pathway. The results indicate that on these SAAs, the nearest host sites are the most effective, where the stabilization effect provided by dopant atoms can be maximized. However, their initial states can be further stabilized by dopant atoms, thereby increasing the energy barriers on these SAAs.

**Figure 19:**
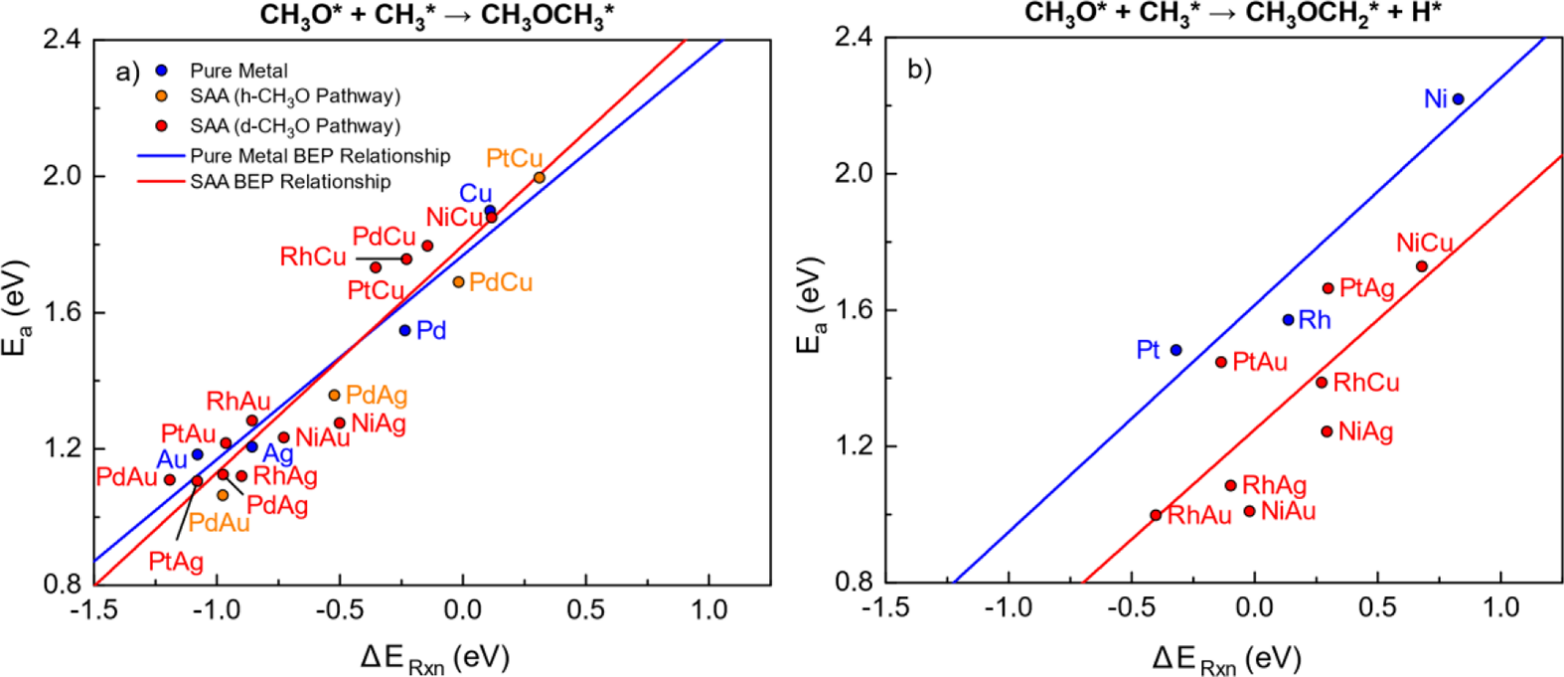
(a) BEP relationships for the h-pathway occurring on group 1 SAAs and pure metal(111) surfaces. (b) BEP relationships for the d-pathway occurring on all kinds of SAA and pure metal(111) surfaces. The plots show the activation energy as a function of the reaction energy. Linear fits are drawn for each reaction on each surface type. Corresponding linear regression fitting parameters and error analysis are given in Table S12.

Finally, BEP relationships for the C–O coupling were obtained by fitting the reaction energy to the activation energy of the h- and d-CH_3_O pathways on each SAA. BEP relationships for the h-pathway occurring on group 1 SAAs and every pure metal(111) surface exhibit *R*^2^ values of 0.910 and 0.951, respectively, indicating that C–O coupling for group 1 SAAs and pure metals exhibit strong linear BEP relations (Figure 19a). After employing Student *t*-tests to determine whether these two linear BEP relationships are different, it is found that linear regressions for group 1 SAAs and pure metal h-pathways have statistically identical slopes and intercepts at the 90% confidence level (Table S12).

On the other hand, the BEP relationship of the h-pathway of group 2 SAAs has an *R*^2^ of 0.587, indicating that the BEP correlation of group 1 catalysts is stronger than that of group 2 (Figure 19b). This might be because the h-pathway on group 2 SAAs involves both dehydrogenation and C–O coupling, resulting in a more complicated concerted reaction. The line fitted to the BEP relationship for SAAs lies below that for pure metals, which means that SAAs exhibit, in general, lower activation energies than pure metals, suggesting that C–O coupling with dehydrogenation occurs more readily on group 2 SAAs.

## 4 Conclusions

In this study, we conducted a thorough investigation of the catalytic performance of SAAs for pertinent reactions. DFT was used to first examine the adsorption behavior of oxygenated organic and inorganic species (including CH_3_OH, CH_3_O, CH_3_OCH_3_, CH_3_OCH_2_, CH_2_OH, CH_2_O, H_2_O, and OH), followed by investigating the mechanisms of several key elementary steps relevant to the decomposition of CH_3_OH and the synthesis of CH_3_OCH_3_ on these catalysts. Finally, BEP relationships for these elementary steps on pure metals and SAAs were developed to reveal the unique catalytic behavior of the SAA materials.

The calculated adsorption energies indicate that for the majority of the adsorbates discussed in this study the formation energies on the SAA surfaces lie between those of their monometallic components, except for Ni/Au(111) and Rh/Au(111). Moreover, the binding site preference of oxygenated organic adsorbates is generally determined by the number of dangling bonds in the adsorbate, thereby being subject to simple adsorption valency rules that are generally followed by those of pure metal surfaces. According to the TCS relations of the formation energy, we find that after replacing the H atom with CH_3_, the formation energy of some simple oxygenates, including CH_3_O, CH_3_OCH_2_, CH_3_OH, and CH_3_OCH_3_ exhibit a strong linear correlation with the formation energy of the original adsorbate (i.e., OH, CH_2_OH, and H_2_O) on both pure metals and SAAs.

Compared with pure host metals, our results show that Ag-based SAAs [except for Pt/Ag(111)] and Pd/Au(111) have the potential to selectively catalyze CH_3_OH dehydrogenation to CH_2_O via the CH_3_O- or CH_2_OH-path, respectively. In addition, by comparing these activities, we determine that SAAs generally exhibit lower apparent activation energies than their pure host metals. Doping Ag(111) with Rh or Ni can significantly increase its activity for CH_3_OH dehydrogenation to CH_2_O. A concerted step that involves C–H activation in CH_3_ and C–O coupling toward DME is found on Ni-, Pt-, and Rh-doped SAAs. In this case, Pd/Au(111) has the lowest apparent activation energy for CH_3_OCH_3_ synthesis without breaking the C–H bond.

Finally, we built BEP relationships for the aforementioned bond dissociations on SAAs and pure metals. For C–H and O–H scission in CH_3_OH and C–H activation in CH_3_O, the SAA BEP relations deviate from and lie below those of pure metals. In this case, for a given reaction energy, the SAAs can exhibit broadly lower activation energies in comparison to the pure metal surfaces. On the other hand, no clear BEP linear relation is found for either SAAs or pure metals for the activation of the O–H bond in CH_2_OH. The BEP line of the concerted C–O coupling step lies below that of C–O coupling without C–H activation, indicating that Ni-, Pt-, and Rh-doped SAAs provide a lower-energy pathway for the C–O coupling by activating the H atoms in CH_3_ during the elementary step. Thus, SAAs combine both weak binding and low activation energy, allowing for optimized catalytic performance.

The wealth of data presented in this article, along with our interpretations, will aid in the discovery and development of SAA materials that exhibit unique and exciting catalytic behavior for oxygenate chemistries relevant to the renewable energy and sustainability fields.

## Supporting Information Available

Additional data, including lattice constants, formation energies of fragments, activation and reaction energies of dissociation reactions, parameters of thermochemical scaling, and BEP relations, and details of Student *t* tests (PDF), DFT-optimized configurations of fragments on pure metals, configurations and distances between H and C atom in CH_3_ and the dopant atom in the transition states of C–O coupling, and apparent activation energy comparison among SAAs (PDF)
