# Supplementary material for: Elucidating the Reactivity of Oxygenates on Single-Atom Alloy Catalysts
Source: ACS Catal. 2023 Nov 27;13(24):15851–68. doi: 10.1021/acscatal.3c03954 (PMC10729050; doi:10.1021/acscatal.3c03954)
Supplement: Supplementary file 1 — cs3c03954_si_001.pdf [file cs3c03954_si_001.pdf]

SUPPLEMENTAL MATERIAL

Elucidating the Reactivity of Oxygenates on Single-Atom Alloy Catalysts

Weitian Li,[1,3] Simran Effricia Madan,[1] Romain Réocreux,[1,2] and Michail Stamatakis[*,1,3]

michail.stamatakis@chem.ox.ac.uk

[1] Thomas Young Centre and Department of Chemical Engineering, University College London, Roberts Building, Torrington Place, London WC1E 7JE, UK.

[2] Yusuf Hamied Department of Chemistry, University of Cambridge, Lensfield Road, CB2 1EW, Cambridge, United Kingdom

[3] Current address: Inorganic Chemistry Laboratory, University of Oxford, S Parks Rd, Oxford, OX1 3QR. United Kingdom

**Metal**	**DFT Lattice Constant (Å)**
Ag	4.077
Au	4.127
Cu	3.608
Ni	3.478
Pd	3.909
Pt	3.961
Rh	3.829

Table S1. Optimized lattice constants from DFT using OptB86b-vdW for FCC Cu, Ag, Au, Ni, Pd, Pt and Rh. The lattice constants are found by performing total energy minimization at various FCC unit cell volumes (a=b=c, α=β=γ=90°), fitting the resulting energies and volumes to a Murnaghan equation of state and taking the minimum thereof.

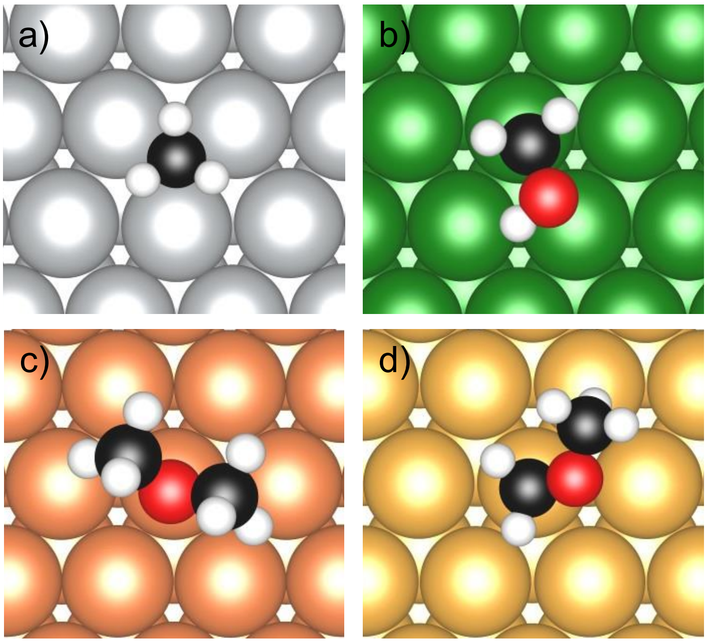

Figure S1. DFT-optimized structures for (a) the CH_3O intermediate on Ag(111), (b) the CH_2OH intermediate on Pd(111), (c) the CH_3OCH_3 intermediate on Cu(111), and (d) the CH_2OCH_3 intermediate on Au(111). Pd in green, Cu in orange, Au in gold, Ag in silver, C in black, O in red, and H in white in the figure.

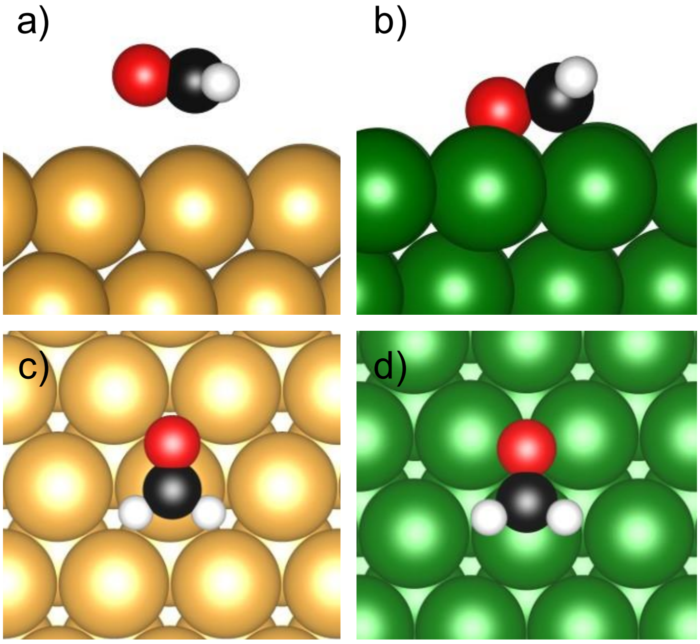

Figure S2. Front views of DFT-optimized structures for the CH_2O intermediates on (a) Au(111) and (b) Pd(111). Top views of DFT-optimized structures for the CH_2O intermediates on (c) Au(111) and (d) Pd(111). Pd in green, Au in gold, C in black, O in red, and H in white in the figure.

	Formation Energy, E_F (eV)							
Surface	CH_3	OH	CH_3O	CH_2OH	CH_3OCH_2	H_2O	CH_3OH	CH_3OCH_3
Ag(111)	1.21	-0.72	0.25	1.02	1.79	-1.33	-0.33	0.55
Au(111)	0.87	-0.14	0.74	0.54	1.32	-1.33	-0.36	0.52
Cu(111)	0.67	-1.28	-0.32	0.64	1.41	-1.41	-0.43	0.44
Ni(111)	0.15	-1.50	-0.66	0.02	0.84	-1.53	-0.57	0.29
Pd(111)	0.35	-0.72	0.11	-0.18	0.69	-1.52	-0.57	0.25
Pt(111)	0.03	-0.44	0.29	-0.45	0.43	-1.56	-0.66	0.21
Rh(111)	0.17	-1.19	-0.41	-0.33	0.50	-1.63	-0.71	0.15
Ni/Ag(111)	0.54	-1.23	-0.27	0.27	1.11	-1.52	-0.55	0.40
Ni/Au(111)	0.63	-0.81	0.09	0.25	1.10	-1.69	-0.73	0.23
Ni/Cu(111)	0.47	-1.37	-0.46	0.22	1.03	-1.52	-0.55	0.35
Pd/Ag(111)	0.77	-0.67	0.29	0.46	1.31	-1.35	-0.36	0.50
Pd/Au(111)	0.72	-0.26	0.63	0.26	1.09	-1.48	-0.51	0.35
Pd/Cu(111)	0.73	-1.02	-0.10	0.41	1.24	-1.41	-0.43	0.43
Pt/Ag(111)	0.42	-0.55	0.37	0.08	0.95	-1.39	-0.40	0.49
Pt/Au(111)	0.28	-0.36	0.53	-0.12	0.73	-1.43	-0.48	0.38
Pt/Cu(111)	0.42	-0.80	0.10	0.09	0.93	-1.43	-0.46	0.44
Rh/Ag(111)	0.37	-0.89	0.03	-0.05	0.84	-1.52	-0.54	0.35
Rh/Au(111)	0.32	-0.66	0.18	-0.17	0.71	-1.70	-0.75	0.15
Rh/Cu(111)	0.46	-1.11	-0.21	0.02	0.87	-1.56	-0.59	0.27

Table S2. Formation energies (E_F) of fragments adsorbed to the most favored (lowest energy) adsorption site on pure metal and SAA (111) surfaces, as computed by DFT. Values of E_F are relative to CH_4(g), H_2(g), and CO_2(g) as given by equation (1) in the main text.

Linear Regression										
Species 1 (x)	Species 2 (y)		a	b (eV)	R^2	MAE (eV)	T-test (90% Confidence)		T-test (99% Confidence)	
							Diff'nt Slope	Diff'nt Intercept	Diff'nt Slope	Diff'nt Intercept
OH	CH_3O	Pure	0.984 ± 0.081	0.843 ± 0.078	0.967	0.073	No	No	No	No
		SAA	0.966 ± 0.029	0.881 ± 0.025	0.991	0.022				
		Global	0.979 ± 0.039	0.867 ± 0.035	0.974	0.046				
CH_2OH	CH_3OCH_2	Pure	0.928 ± 0.014	0.833 ± 0.007	0.999	0.015	No	Yes	No	No
		SAA	0.928 ± 0.030	0.861 ± 0.007	0.990	0.015				
		Global	0.839 ± 0.006	0.926 ± 0.015	0.995	0.017				
H_2O	CH_3OH	Pure	1.241 ± 0.065	1.313 ± 0.096	0.986	0.014	Yes	Yes	No	No
		SAA	1.088 ± 0.026	1.105 ± 0.039	0.995	0.006				
		Global	1.135 ± 0.038	1.163 ± 0.057	0.981	0.013				
CH_3OH	CH_3OCH_3	Pure	1.073 ± 0.053	0.899 ± 0.028	0.988	0.012	No	No	No	No
		SAA	0.842 ± 0.026	0.929 ± 0.050	0.977	0.014				
		Global	0.932 ± 0.060	0.845 ± 0.032	0.934	0.023				
H_2O	CH_3OCH_3	Pure	1.336 ± 0.078	2.315 ± 0.115	0.983	0.018	Yes	Yes	Yes	Yes
		SAA	0.898 ± 0.088	1.710 ± 0.133	0.912	0.024				
		Global	1.038 ± 0.092	1.893 ± 0.137	0.882	0.031				

Table S3. Thermo-chemical scaling relations (TCS) for species 1 (x-axis) and species 2 (y-axis) adsorbed in their most favored adsorption sites on pure metals and SAAs. Linear Regression parameters (slope, a and intercept, b in eV) are given in addition to coefficients of determination (R^2) and mean absolute errors in eV (MAE). Results of Student's T-test (to determine if the slopes and intercepts of pure metal and SAA TCS relations are different) are given for 90 % and 99 % confidence intervals as "Yes" or "No" for different and not different, respectively. Details of calculation methods can be found in the work of Darby et al.[1]

$CH_3OH^* \rightarrow CH_2OH^* + H^*$							
Surface	h-CH_3OH pathway		d-CH_3OH pathway		Preferable pathway	Energy difference	
	E_a (eV)	E_{Rxn} (eV)	E_a (eV)	E_{Rxn} (eV)		ΔE^{IS}_{Tot} (eV)	ΔE^{TS}_{Tot} (eV)
Ag(111)	1.91	1.67	--	--	--	--	--
Au(111)	1.52	1.20	--	--	--	--	--
Cu(111)	1.40	0.81	--	--	--	--	--
Ni(111)	0.78	0.07	--	--	--	--	--
Pd(111)	0.64	0.03	--	--	--	--	--
Pt(111)	0.63	-0.25	--	--	--	--	--
Rh(111)	0.67	0.01	--	--	--	--	--
Ni/Ag(111)	1.51	0.54	0.94	0.85	d-CH_3OH	0.21	0.79
Ni/Au(111)	1.34	0.80	1.06	1.00	d-CH_3OH	0.40	0.68
Ni/Cu(111)	1.25	0.63	0.88	0.55	d-CH_3OH	0.12	0.49
Pd/Ag(111)	1.63	1.42	1.10	0.93	d-CH_3OH	0.00	0.52
Pd/Au(111)	1.33	0.91	1.07	0.85	d-CH_3OH	0.17	0.43
Pd/Cu(111)	1.38	0.82	1.09	0.73	d-CH_3OH	-0.03	0.27
Pt/Ag(111)	1.49	1.14	0.81	0.44	d-CH_3OH	-0.12	0.56
Pt/Au(111)	1.25	0.55	0.78	0.39	d-CH_3OH	0.11	0.59
Pt/Cu(111)	1.35	0.81	0.88	0.39	d-CH_3OH	-0.13	0.35
Rh/Ag(111)	1.37	0.00	0.59	0.32	d-CH_3OH	0.20	0.98
Rh/Au(111)	1.19	0.58	0.70	0.45	d-CH_3OH	0.42	0.92
Rh/Cu(111)	1.22	0.58	0.69	0.33	d-CH_3OH	0.18	0.71

Table S4. Activation energies (Ea) and reaction energies (ΔE_{Rxn}) for the h-CH_3OH and the d-CH_3OH C-H dissociation reaction pathways on pure metal (blue) and SAA (red) surfaces. Preferable pathway in the table is defined as the pathway with lower absolute energy of the transition state. Energy differences of ΔE^{IS}_{Tot} and ΔE^{TS}_{Tot} are calculated by the activation energy of h-pathway minus that of the d-pathway and the reaction energy of h-CH_3OH pathway minus that of the d-CH_3OH pathway, respectively. Double dash means that we were not able to find a transition state for the corresponding pathway.

Surface	h-CH_3OH pathway		d-CH_3OH pathway		Preferable pathway	Energy difference	
	E_a (eV)	E_{Rxn} (eV)	E_a (eV)	E_{Rxn} (eV)		ΔE^{IS}_{Tot} (eV)	ΔE^{TS}_{Tot} (eV)
**$CH_3OH^* \rightarrow CH_3O^* + H^*$**							
Ag(111)	1.53	0.84	--	--	--	--	--
Au(111)	1.72	1.31	--	--	--	--	--
Cu(111)	1.05	-0.11	--	--	--	--	--
Ni(111)	0.84	-0.50	--	--	--	--	--
Pd(111)	0.96	0.15	--	--	--	--	--
Pt(111)	0.73	0.47	--	--	--	--	--
Rh(111)	0.81	-0.08	--	--	--	--	--
Ni/Ag(111)	1.05	0.35	0.96	0.19	d-CH_3OH	0.21	0.29
Ni/Au(111)	1.43	0.43	1.21	0.70	d-CH_3OH	0.40	0.62
Ni/Cu(111)	0.87	-0.26	0.96	-0.23	d-CH_3OH	0.12	0.04
Pd/Ag(111)	1.16	0.54	1.43	0.64	h-CH_3OH	0.00	-0.28
Pd/Au(111)	1.57	1.05	1.58	1.21	d-CH_3OH	0.17	0.16
Pd/Cu(111)	0.86	-0.07	1.39	-0.15	h-CH_3OH	-0.03	-0.56
Pt/Ag(111)	0.87	0.41	1.24	0.54	h-CH_3OH	-0.12	-0.49
Pt/Au(111)	1.15	1.02	1.34	1.09	h-CH_3OH	0.11	-0.08
Pt/Cu(111)	0.72	-0.07	1.30	-0.19	h-CH_3OH	-0.13	-0.71
Rh/Ag(111)	0.84	0.28	0.96	0.20	d-CH_3OH	0.20	0.08
Rh/Au(111)	1.27	0.44	1.15	0.85	d-CH_3OH	0.42	0.54
Rh/Cu(111)	0.68	-0.27	1.05	-0.01	h-CH_3OH	0.18	-0.19

Table S5. Activation energies (Ea) and reaction energies (ΔE_{Rxn}) for the h-CH_3OH and the d-CH_3OH O-H dissociation reaction pathways on pure metal (blue) and SAA (red) surfaces. The "preferable pathway" in this table is defined as the pathway with the lower absolute energy of the transition state. Energy differences of ΔE^{IS}_{Tot} and ΔE^{TS}_{Tot} are calculated by the activation energy of h-pathway minus that of the d-pathway and the reaction energy of h-CH_3OH pathway minus that of the d-CH_3OH pathway, respectively. Double dash means that we were not able to find a transition state for the corresponding pathway.

Surface	$CH_3O^* \rightarrow CH_2O^* + H^*$						
	h-CH_3O pathway		d-CH_3O pathway		Preferable pathway	Energy difference	
	E_a (eV)	E_{Rxn} (eV)	E_a (eV)	E_{Rxn} (eV)		ΔE^{IS}_{Tot} (eV)	ΔE^{TS}_{Tot} (eV)
Ag(111)	1.16	0.99	--	--	--	--	--
Au(111)	0.76	0.45	--	--	--	--	--
Cu(111)	1.22	0.89	--	--	--	--	--
Ni(111)	0.86	0.38	--	--	--	--	--
Pd(111)	0.60	-0.23	--	--	--	--	--
Pt(111)	0.18	-0.48	--	--	--	--	--
Rh(111)	0.57	-0.10	--	--	--	--	--
Ni/Ag(111)	1.24	1.07	0.74	0.56	d-CH_3O	--*	0.51
Ni/Au(111)	0.69	0.63	0.79	0.57	d-CH_3O	0.14	0.04
Ni/Cu(111)	1.22	0.55	0.76	0.47	d-CH_3O	--*	0.46
Pd/Ag(111)	1.11	0.87	0.78	0.45	d-CH_3O	--*	0.33
Pd/Au(111)	--	--	0.72	0.34	d-CH_3O	0.19	--
Pd/Cu(111)	1.06	0.80	1.00	0.46	d-CH_3O	--*	0.06
Pt/Ag(111)	0.98	0.77	0.45	0.33	d-CH_3O	0.12	0.65
Pt/Au(111)	0.75	0.44	--	--	h-CH_3O	-0.11	--
Pt/Cu(111)	1.05	0.75	0.73	0.15	d-CH_3O	--*	0.32
Rh/Ag(111)	--	--	0.42	-0.05	d-CH_3O	0.06	--
Rh/Au(111)	0.77	0.38	--	--	h-CH_3O	-0.23	--
Rh/Cu(111)	1.04	0.74	0.56	-0.02	d-CH_3O	--*	0.48

Table S6. Activation energies (Ea) and reaction energies (ΔE_{Rxn}) for the h-CH_3O and the d-CH_3O C-H dissociation reaction pathways on pure metal (blue) and SAA (red) surfaces. Preferable pathway in the table is defined as the pathway with lower absolute energy of the transition state. Energy differences of ΔE^{IS}_{Tot} and ΔE^{TS}_{Tot} are calculated by the activation energy of h-pathway minus that of the d-pathway and the reaction energy of h-CH_3OH pathway minus that of the d-CH_3OH pathway, respectively. Double dash means that we were not able to find a transition state for the corresponding pathway.

$CH_2OH^* \rightarrow CH_2O^* + H^*$							
Surface	h-CH_2OH pathway		d-CH_2OH pathway		Preferable pathway	Energy difference	
	E_a (eV)	E_{Rxn} (eV)	E_a (eV)	E_{Rxn} (eV)		ΔE_{Tot}^{IS} (eV)	ΔE_{Tot}^{TS} (eV)
Ag(111)	0.98	0.22	--	--	--	--	--
Au(111)	0.87	0.65	--	--	--	--	--
Cu(111)	0.94	0.00	--	--	--	--	--
Ni(111)	0.65	-0.32	--	--	--	--	--
Pd(111)	0.72	0.03	--	--	--	--	--
Pt(111)	0.64	0.34	--	--	--	--	--
Rh(111)	0.71	-0.15	--	--	--	--	--
Ni/Ag(111)	0.55	-0.30	1.38	0.24	h-CH_2OH	0.74	-0.09
Ni/Au(111)	0.82	0.29	1.05	0.49	d-CH_2OH	0.30	0.06
Ni/Cu(111)	0.78	-0.16	0.99	-0.10	d-CH_2OH	0.44	0.24
Pd/Ag(111)	0.57	-0.13	1.26	0.45	h-CH_2OH	0.54	-0.15
Pd/Au(111)	0.75	0.35	--	--	h-CH_2OH	0.29	--
Pd/Cu(111)	0.86	0.05	0.90	0.08	d-CH_2OH	0.23	0.18
Pt/Ag(111)	0.29	-0.32	1.48	0.75	h-CH_2OH	0.90	-0.28
Pt/Au(111)	0.44	0.07	1.43	1.12	h-CH_2OH	0.65	-0.34
Pt/Cu(111)	0.61	0.03	1.05	0.30	d-CH_2OH	0.55	0.11
Rh/Ag(111)	0.32	-0.46	1.42	0.45	h-CH_2OH	1.08	-0.02
Rh/Au(111)	0.54	0.12	--	--	h-CH_2OH	0.73	--
Rh/Cu(111)	0.62	-0.77	1.00	0.11	d-CH_2OH	0.68	0.29

Table S7. Activation energies (Ea) and reaction energies (ΔE_{Rxn}) for the h-CH_2OH and the d-CH_2OH O-H dissociation reaction pathways on pure metal (blue) and SAA (red) surfaces. Preferable pathway in the table is defined as the pathway with lower absolute energy of the transition state. Energy differences of ΔE_{Tot}^{IS} and ΔE_{Tot}^{TS} are calculated by the activation energy of h-pathway minus that of the d-pathway and the reaction energy of h-CH_3OH pathway minus that of the d-CH_3OH pathway, respectively. Double dash means that we were not able to find a transition state for the corresponding pathway.

CH_3O^*(dopant) + CH_3^* → $CH_3OCH_3^*$ / CH_3O^*(host) + CH_3^* → $CH_3OCH_2^*$ + H^*							
Group 1	h-CH_3OH pathway		d-CH_3OH pathway		Preferable	Energy difference	
surface	E_a (eV)	E_{Rxn} (eV)	E_a (eV)	E_{Rxn} (eV)	pathway	ΔE^{IS}_{Tot} (eV)	ΔE^{TS}_{Tot} (eV)
Ag(111)	1.21	-0.86	--	--	--	--	--
Au(111)	1.18	-1.08	--	--	--	--	--
Cu(111)	1.90	0.11	--	--	--	--	--
Pd(111)	1.55	-0.23	--	--	--	--	--
Pd/Ag(111)	1.36	-0.52	1.11	-1.19	h-CH_3OH	-0.46	-0.23
Pd/Au(111)	1.06	-0.98	1.80	-0.14	h-CH_3OH	-0.07	-0.11
Pd/Cu(111)	1.69	-0.02	1.11	-1.08	h-CH_3OH	-0.14	-0.24
Pt/Cu(111)	2.00	0.31	1.73	-0.35	h-CH_3OH	-0.59	-0.33

Table S8. Activation energies (Ea) and reaction energies (ΔE_{Rxn}) for the h-CH_3O and the d-CH_3O C-O coupling reaction pathways on Group 1 pure metal (blue) and SAA (red) surfaces. Preferable pathway in the table is defined as the pathway with lower absolute energy of the transition state. Energy differences of ΔE^{IS}_{Tot} and ΔE^{TS}_{Tot} are calculated by the activation energy of h-pathway minus that of the d-pathway and the reaction energy of h-CH_3OH pathway minus that of the d-CH_3OH pathway, respectively. Double dash means that we were not able to find a transition state for the corresponding pathway.

CH_3O^*(dopant/host) + CH_3^* → $CH_3OCH_3^*$							
Group 2	h-CH_3OH pathway		d-CH_3OH pathway		Preferable	Energy difference	
surface	E_a (eV)	E_{Rxn} (eV)	E_a (eV)	E_{Rxn} (eV)	pathway	ΔE_{Tot}^{IS} (eV)	ΔE_{Tot}^{TS} (eV)
Ni(111)	2.22	0.83	--	--	--	--	--
Pt(111)	1.48	-0.32	--	--	--	--	--
Rh(111)	1.57	0.14	--	--	--	--	--
Ni/Ag(111)	1.24	0.29	1.28	-0.50	h-CH_3OH	-0.15	-0.18
Ni/Au(111)	1.01	-0.02	1.23	-0.73	d-CH_3OH	0.39	0.16
Ni/Cu(111)	1.73	0.68	1.88	0.12	h-CH_3OH	-0.09	-0.25
Pt/Ag(111)	1.66	0.30	1.11	-1.08	h-CH_3OH	-0.89	-0.34
Pt/Au(111)	1.45	-0.14	1.22	-0.96	h-CH_3OH	-0.30	-0.07
Rh/Ag(111)	1.08	-0.10	1.12	-0.90	h-CH_3OH	-0.59	-0.62
Rh/Au(111)	1.00	-0.40	1.28	-0.86	h-CH_3OH	0.10	-0.18
Rh/Cu(111)	1.39	0.27	1.76	-0.23	h-CH_3OH	-0.27	-0.64

Table S9. Activation energies (Ea) and reaction energies (ΔE_{Rxn}) for the h-CH_3O and the d-CH_3O C-O coupling reaction pathways on Group 2 pure metal (blue) and SAA (red) surfaces. Preferable pathway in the table is defined as the pathway with lower absolute energy of the transition state. Energy differences of ΔE_{Tot}^{IS} and ΔE_{Tot}^{TS} are calculated by the activation energy of h-pathway minus that of the d-pathway and the reaction energy of h-CH_3OH pathway minus that of the d-CH_3OH pathway, respectively. Double dash means that we were not able to find a transition state for the corresponding pathway.

Surface	**Formation energy:**	$CH_3O^* + CH_3^* \rightarrow CH_3OCH_3^*$	/	$CH_3O^* + CH_3^* \rightarrow CH_3OCH_2^* + H^*$		
		h-CH_2OH pathway			d-CH_2OH pathway	
	$E_{F,ini}$ (eV)	$E_{F,tra}$ (eV)	$E_{F,fin}$ (eV)	$E_{F,ini}$ (eV)	$E_{F,tra}$ (eV)	$E_{F,fin}$ (eV)
Ag(111)	1.41	2.62	0.55	--	--	--
Au(111)	1.60	2.78	0.52	--	--	--
Cu(111)	0.33	2.23	0.44	--	--	--
Ni(111)	-0.45	1.77	0.38	--	--	--
Pd(111)	0.49	2.04	0.25	--	--	--
Pt(111)	0.34	1.82	0.02	--	--	--
Rh(111)	-0.17	1.41	-0.03	--	--	--
Ni/Ag(111)	0.75	2.00	1.04	0.90	2.17	0.40
Ni/Au(111)	1.35	2.36	1.32	0.96	2.19	0.23
Ni/Cu(111)	0.14	1.86	0.81	0.23	2.11	0.35
Pd/Ag(111)	1.01	2.37	0.49	1.47	2.60	0.50
Pd/Au(111)	1.47	2.54	0.50	1.54	2.65	0.35
Pd/Cu(111)	0.45	2.14	0.43	0.59	2.38	0.44
Pt/Ag(111)	0.67	2.34	0.97	1.57	2.67	0.49
Pt/Au(111)	1.05	2.50	0.91	1.35	2.56	0.38
Pt/Cu(111)	0.20	2.20	0.51	0.79	2.53	0.44
Rh/Ag(111)	0.66	1.75	0.56	1.25	2.37	0.35
Rh/Au(111)	1.11	2.11	0.71	1.01	2.29	0.15
Rh/Cu(111)	0.24	1.62	0.51	0.50	2.26	0.27

Table S10. Formation energies of initial states ($E_{F,ini}$), transition states ($E_{F,tra}$), and final states ($E_{F,fin}$) of C-O coupling on pure metal and SAA (111) surfaces, as computed by DFT. Values of E_F are relative to CH_4(g), H_2(g), and CO_2(g) as given by equation (1) in the main text. Double dash means that we were not able to find a transition state for the corresponding pathway.

Formation energy: $CH_2OH^* \rightarrow CH_2O^* + H^*$						
Surface	h-CH_2OH pathway			d-CH_2OH pathway		
	$E_{F,ini}$ (eV)	$E_{F,tra}$ (eV)	$E_{F,fin}$ (eV)	$E_{F,ini}$ (eV)	$E_{F,tra}$ (eV)	$E_{F,fin}$ (eV)
Ag(111)	1.01	1.99	1.23	--	--	--
Au(111)	0.53	1.40	1.18	--	--	--
Cu(111)	0.63	1.57	0.63	--	--	--
Ni(111)	0.01	0.66	-0.31	--	--	--
Pd(111)	-0.18	0.54	-0.14	--	--	--
Pt(111)	-0.45	0.19	-0.11	--	--	--
Rh(111)	-0.34	0.37	-0.49	--	--	--
Ni/Ag(111)	1.00	1.54	0.69	0.26	1.63	0.50
Ni/Au(111)	0.54	1.35	0.82	0.24	1.29	0.72
Ni/Cu(111)	0.65	1.44	0.49	0.21	1.20	0.11
Pd/Ag(111)	0.99	1.56	0.85	0.45	1.71	0.90
Pd/Au(111)	0.53	1.29	0.89	0.25	--	--
Pd/Cu(111)	0.62	1.48	0.68	0.40	1.30	0.47
Pt/Ag(111)	0.97	1.26	0.64	0.07	1.54	0.82
Pt/Au(111)	0.52	0.96	0.59	-0.13	1.29	0.98
Pt/Cu(111)	0.62	1.23	0.66	0.07	1.12	0.38
Rh/Ag(111)	1.02	1.34	0.55	-0.06	1.36	0.39
Rh/Au(111)	0.55	1.09	0.66	-0.19	--	--
Rh/Cu(111)	0.69	1.31	-0.08	0.01	1.01	0.12

Table S11. Formation energies of initial states ($E_{F,ini}$), transition states ($E_{F,tra}$), and final states ($E_{F,fin}$) of CH_2OH O-H activation on pure metal and SAA (111) surfaces, as computed by DFT. Values of E_F are relative to CH_4(g), H_2(g), and CO_2(g) as given by equation (1) in the main text. Double dash means that we were not able to find a transition state for the corresponding pathway.

Reaction	Surface	Linear Regression a	b (eV)	R^2	MAE (eV)	T-test (90% Confidence) Diff'nt Slope	Diff'nt Intercept
$CH_3OH^* \rightarrow CH_2OH^* + H^*$	Pure	0.712 ± 0.047	0.720 ± 0.039	0.979	0.066	No	Yes
	SAA	0.614 ± 0.103	0.509 ± 0.067	0.780	0.060		
	Global	0.664 ± 0.080	0.577 ± 0.058	0.800	0.130		
$CH_3OH^* \rightarrow CH_3O^* + H^*$	Pure	0.499 ± 0.161	0.943 ± 0.104	0.658	0.090	No	No
	SAA	0.443 ± 0.068	0.850 ± 0.041	0.809	0.172		
	Global	0.462 ± 0.075	0.884 ± 0.046	0.693	0.124		
$CH_3O^* \rightarrow CH_2O^* + H^*$	Pure	0.621 ± 0.078	0.595 ± 0.046	0.928	0.080	No	No
	SAA	0.521 ± 0.182	0.530 ± 0.072	0.452	0.080		
	Global	0.589 ± 0.080	0.543 ± 0.038	0.762	0.093		
$CH_2OH^* \rightarrow CH_2O^* + H^*$	Pure	0.163 ± 0.180	0.770 ± 0.058	0.141	0.099	No	No
	SAA	0.698 ± 0.222	0.694 ± 0.062	0.497	0.024		
	Global	0.491 ± 0.158	0.711 ± 0.046	0.363	0.164		
$CH_3O^* + CH_3^* \rightarrow CH_3OCH_3^*$	Pure	0.599 ± 0.096	1.768 ± 0.067	0.951	0.063	No	No
	SAA	0.667 ± 0.056	1.797 ± 0.040	0.910	0.080		
	Global	0.652 ± 0.047	1.791 ± 0.033	0.916	0.076		
$CH_3O^* + CH_3^* \rightarrow CH_3OCH_2^* + H^*$	Pure	0.666 ± 0.203	1.615 ± 0.105	0.915	0.090	No	Yes
	SAA	0.644 ± 0.022	1.250 ± 0.074	0.587	0.140		
	Global	0.710 ± 0.203	1.341 ± 0.080	0.576	0.201		

Table S12. Linear regression slopes (a), intercepts (b, in eV), R^2 and MAE in eV for the BEP relationship for six reactions; C-H and O-H activation of CH_3OH, C-H activation of CH_3O, C-H activation of CH_2OH, C-O coupling between CH_3 and CH_3O. Data is given for these reactions on pure metal (111) surfaces (blue), SAA (111) surfaces (red) and globally (black). Results of Student's T-test (to determine if the slopes and intercepts of pure metal and SAA BEP relations are different) are given for 90 % confidence intervals as "Yes" or "No" for different and not different, respectively.

**Surface**	**$D_{H\text{-dopant}}$ (Å)**	**$D_{C\text{-dopant}}$ (Å)**
Group 1 Pure metal and SAA		
Ag(111)	2.42	2.61
Au(111)	2.38	2.54
Cu(111)	2.27	2.52
Pd(111)	1.99	2.33
Pd/Ag(111)	2.04	2.27
Pd/Au(111)	2.09	2.27
Pd/Cu(111)	2.09	2.41
Pt/Cu(111)	2.08	2.43
Group 2 Pure metal and SAA		
Ni(111)	1.64	1.96
Pt(111)	1.79	2.06
Rh(111)	1.75	2.02
Ni/Ag(111)	1.64	1.92
Ni/Au(111)	1.74	1.95
Ni/Cu(111)	1.63	1.93
Pt/Ag(111)	1.87	2.15
Pt/Au(111)	1.95	2.18
Rh/Ag(111)	1.75	2.01
Rh/Au(111)	1.81	2.03
Rh/Cu(111)	1.73	2.04

Table S13. Distance between the lowest H atom in CH_3 and the dopant atom ($D_{H\text{-dopant}}$) and distance between the C atom in CH_3 and the dopant atom ($D_{C\text{-dopant}}$) on the slab for Group 1 SAAs (blue) and Group 2 SAAs (red) in transition states of C-O coupling h-CH_3O pathway.

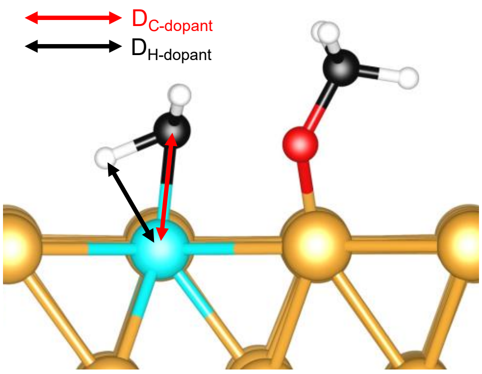

Figure S3. Transition states of C-O coupling h-CH_3O pathway on Ni/Au(111). Distance between the lowest H atom in CH_3 and the dopant atom ($D_{H\text{-dopant}}$) and distance between the C atom in CH_3 and the dopant atom ($D_{C\text{-dopant}}$) are show in the black and the red arrow, respectively.

Comparing Apparent Activation Energies among SAAs

As two different pathways are investigated for each step on SAAs (d- and h- pathways), the apparent activation energies on these materials are calculated to properly compare activities among SAAs (Figure S4). Regarding the CH_3OH C-H activation, Rh-doped SAAs have the lowest energy barriers compared to other transition metal doped counterparts. The smallest apparent activation energy among them is observed on Rh/Ag(111), 0.59 eV, which is approximately half of the maximum energy of 1.11 eV, exhibited on Pd/Cu(111), as shown in Figure S4a. Moreover, Pd-doped SAAs exhibit the highest activation energies for CH_3OH C-H scission in SAAs. On the other hand, the opposite trend is found for the CH_2OH O-H activation. In particular, SAAs doped with Pd exhibit lower energy barriers for this dehydrogenation reaction, while Rh-doped SAAs exhibit higher energy barriers compared to other SAAs, except for Rh/Cu(111) (Figure S4b). The data also indicates that Ag-based SAAs generally have the highest activation energies, followed by Au- and Cu-based SAAs.

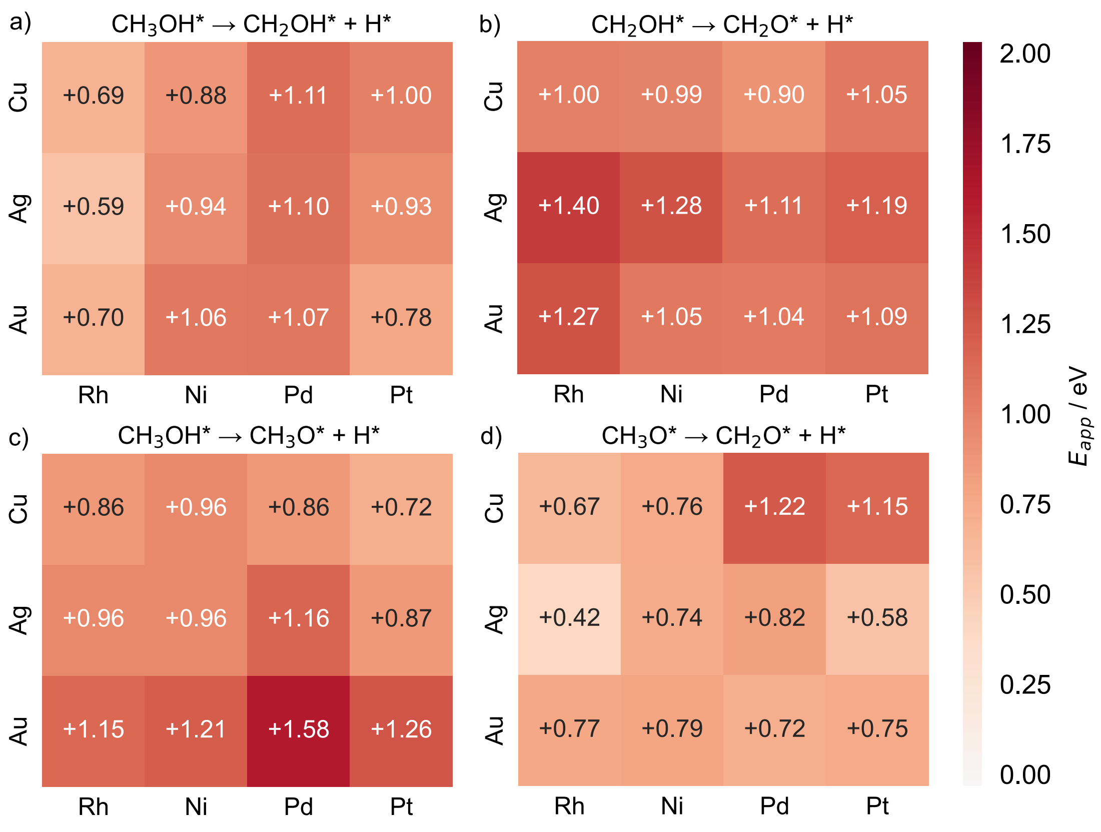

Figure S4. Heatmap charts of the apparent activation energies (E_{app}) for (a) CH_3OH C-H activation, (b) CH_2OH O-H activation, (c) CH_3OH O-H activation, and (d) CH_3O C-H activation for Cu-, Ag- and Au-based SAAs. For each SAA, E_{app} is calculated as the formation energy of the lower-energy transition state (between the d- and h-pathways) minus the energy of the most stable initial state.

Continuing our discussion with the CH_3OH O-H activations, our results indicate that these are less likely to occur on Ag- and Au-based SAAs and are most likely to occur on Cu-based SAAs (Figure S4c). Pd/Au(111) exhibits the highest apparent activation energy (1.58 eV), which is more than twice the lowest energy on Pt/Cu(111) (0.72 eV). As for the CH_3O C-H activation, Pd/Cu(111) and Pt/Cu(111) have large energy barriers at 1.22 and 1.15 eV, respectively, while the lowest energy barrier was observed on Rh/Ag(111) (Figure S4d).

These apparent activation energies can be used to determine the path through which formaldehyde is generated. As Rh/Ag(111) has quite a large energy barrier for the O-H activation of CH_2OH (0.44 and 0.98 eV higher than CH_3OH O-H activation, and CH_3O C-H activation, respectively), the CH_2O species is more likely to be produced through the "CH_3O-path" (Figure S4). This result is also true for Ni/Ag(111) and Pt/Ag(111). On the other hand, because of the high energy barrier of CH_3OH O-H activation on Pd/Au(111), this alloy can preferably generate CH_2OH through the "CH_2OH-path". Although Pd/Cu(111) and Pt/Cu(111) also have high energy barriers for CH_3O C-H activation, similar activation energies are also found in CH_3OH C-H and CH_2OH O-H activations. In conclusion, Ag-based SAAs (except for Pt/Ag(111)), and Pd/Au(111) have the potential to catalyze CH_3OH dehydrogenation to CH_2O via CH_3O- or CH_2OH-path, respectively.

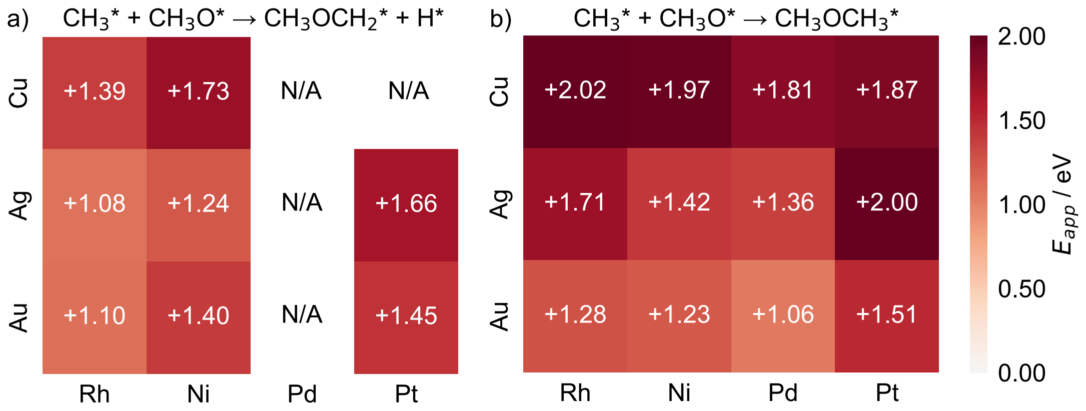

Figure S5. Heatmap charts of the apparent activation energies (E_{app}) for (a) CH_3OCH_2 formation and (b) CH_3OCH_3 formation for Cu-, Ag- and Au-based SAAs. For each SAA, E_{app} is calculated as the formation energy of the lower-energy transition state (between the d- and h-pathways) minus the energy of the most stable initial state.

Moving on to C-O coupling between CH_3 and CH_3O, all SAAs can be divided into two groups; dopant atoms in Group 1 SAAs, which include Pd/Au, Pd/Ag, Pd/Cu, and Pt/Cu, produce CH_3OCH_3 from both host-based and dopant-based initial states. For Group 2 SAAs, the dopant atoms have the potential to activate the C-H bonds in CH_3 along the h-pathway to produce CH_3OCH_2 and H adatoms. Among Group 2 SAAs, only

Ni/Au(111) has a reduced apparent activation energy for CH_3OCH_3 formation, which is 0.17 eV lower than the C-O coupling step towards CH_3OCH_2 (Figure S5). To avoid forming CH_3OCH_2, Pt/Cu(111) and Pd-doped SAAs could be used, which are predicted to be the most promising catalysts for CH_3OCH_3 synthesis. Among these SAAs, Pd/Au(111) has the lowest apparent activation energy, showing the best activity compared to other SAAs. As a “second choice” alternative, Pd/Ag(111) can be considered. Although the apparent activation energy on Pd/Ag(111) is 0.30 eV higher than that of gold, using silver as the host metal can significantly reduce costs.

References

(1) Darby, M. T.; Réocreux, R.; Sykes, E. C. H.; Michaelides, A.; Stamatakis, M. Elucidating the stability and reactivity of surface intermediates on single-atom alloy catalysts. *ACS Catal.* **2018**, *8* (6), 5038-5050.
